# FGF2 as a Potential Tumor Suppressor in Lung Adenocarcinoma

**DOI:** 10.3390/diagnostics16020250

**Published:** 2026-01-13

**Authors:** Shih-Sen Lin, Hsin-Ying Lu, Tsung-Ming Chang, Ying-Sui Sun, Ju-Fang Liu

**Affiliations:** 1 Division of Chest Medicine, Department of Internal Medicine, Shin Kong Wu Ho-Su Memorial Hospital, Taipei 111045, Taiwan; m008932@ms.skh.org.tw; 2 Department of Surgery, School of Medicine, College of Medicine, Taipei Medical University, Taipei 110301, Taiwan; hsinyinglu110@tmu.edu.tw; 3 Division of Cardiovascular Surgery, Department of Surgery, Wan Fang Hospital, Taipei Medical University, Taipei 110301, Taiwan; 4 School of Dental Technology, College of Oral Medicine, Taipei Medical University, Taipei 110301, Taiwan; a03441@tmu.edu.tw (T.-M.C.); yingsuisun@tmu.edu.tw (Y.-S.S.); 5 Translational Medicine Center, Shin Kong Wu Ho-Su Memorial Hospital, Taipei 111045, Taiwan; 6 School of Oral Hygiene, College of Oral Medicine, Taipei Medical University, Taipei 110301, Taiwan; 7 Department of Medical Research, China Medical University Hospital, China Medical University, Taichung 404328, Taiwan

**Keywords:** lung adenocarcinoma (LUAD), fibroblast growth factor 2 (FGF2), bioinformatic analysis, differentially expressed genes (DEGs), metastasis

## Abstract

**Background/Objectives:** Lung adenocarcinoma (LUAD), the predominant subtype of non-small cell lung cancer (NSCLC), is frequently diagnosed at advanced stages with distant metastasis, underscoring the need for effective prognostic biomarkers. Fibroblast growth factor 2 (FGF2), a multifunctional regulator, has shown to play contradictory roles in cancer progression. **Methods:** We analyzed three independent Gene Expression Omnibus (GEO) datasets (GSE19804, GSE18842, and GSE19188) to identify consistently dysregulated genes in LUAD. Functional enrichment (GO, KEGG, and cancer hallmark analysis), protein–protein interaction (PPI) network construction, and hub gene prioritization were performed using public bioinformatic tools. Survival analyses were conducted via the Kaplan–Meier Plotter. The expression of FGF2 was validated across multiple platforms, including TCGA, CPTAC, TNMplot, LCE, and the Human Protein Atlas. Functional assays (Transwell migration and wound healing) demonstrated that exogenous FGF2 significantly suppressed LUAD cell motility in vitro. **Results:** A total of 949 differentially expressed genes (DEGs) were commonly identified across datasets, with enrichment in cell adhesion and metastasis-related pathways. Among the 11 hub genes identified, FGF2 was consistently downregulated in LUAD tissues across all datasets and stages. Higher FGF2 expression was associated with longer overall and progression-free survival. In vitro, FGF2 treatment significantly suppressed the migration and wound healing abilities of LUAD cell lines. **Conclusions:** FGF2 is downregulated in LUAD and inversely associated with metastatic progression and poor prognosis. The observed reduction in cancer cell motility upon FGF2 treatment in vitro, together with its expression pattern, supports a potential tumor-suppressive role and suggests that FGF2 may serve as a candidate non-invasive biomarker for monitoring LUAD metastasis.

## 1. Introduction

Lung cancer remains the leading cause of cancer-related mortality worldwide and is primarily classified into small cell lung cancer (SCLC) and non-small cell lung cancer (NSCLC), the latter accounting for approximately 85% of all cases [1]. NSCLC can be further subdivided into adenocarcinoma, squamous cell carcinoma, and large cell carcinoma [2]. Standard treatment approaches for NSCLC include surgery, chemotherapy, and radiotherapy, either as monotherapy or in combination, depending on disease stage [3]. In recent years, targeted therapies and immunotherapies have significantly improved clinical outcomes for a subset of patients [4]. However, the prognosis for advanced-stage NSCLC remains poor due to late diagnosis, metastasis, drug resistance, and the limited availability of effective therapeutic targets [5].

Metastasis represents a pivotal inflection point in the clinical management of lung cancer, drastically worsening prognosis and complicating therapy; while localized disease can often be controlled with surgery, radiation, or targeted agents, dissemination to distant sites substantially reduces success rates [6]. NSCLC and SCLC display distinct metastatic patterns and survival outcomes, reflecting their biological heterogeneity. The metastatic cascade is driven by a multifaceted interplay of factors—pathological subtype; driver mutations, such as those in the epidermal growth factor receptor; treatment history; and cellular/molecular programs including epithelial-to-mesenchymal transition, extracellular matrix remodeling, angiogenesis, and immune evasion—which together govern dissemination and colonization [7,8,9,10,11]. Prior work has highlighted the importance of understanding these processes, yet systematic analyses of metastatic sites, rates, risk factors, and survival impact remain incomplete. Despite progress in precision medicine, many NSCLC patients continue to experience disease progression via metastasis, underscoring an urgent need to discover and validate novel biomarkers and therapeutic targets—preferably through minimally invasive means like liquid biopsy—to better predict and suppress metastatic potential [12,13].

Fibroblast growth factor 2 (FGF2), also known as basic FGF (bFGF), is a member of the FGF family involved in cell proliferation, differentiation, angiogenesis, and tissue repair [14]. FGF2 exerts its biological effects through interaction with FGF receptors (FGFRs), activating key signaling pathways such as Ras-mitogen-activated protein kinase and phosphoinositide 3-kinase (PI3K)-Akt [15]. Although FGF2 has traditionally been associated with pro-tumorigenic functions—such as promoting angiogenesis and tumor cell survival—emerging evidence suggests a more complex role. In certain contexts, FGF2 has been shown to exert tumor-suppressive effects. For instance, FGF2 can induce cellular senescence in oncogene-amplified cells, suppress proliferation in FGFR1-amplified cancers, and inhibit tumor invasion in gastric cancer models [15,16,17,18,19,20,21]. The dual role of FGF2 in cancer biology highlights the need for context-specific investigation, particularly in NSCLC, where its function remains incompletely understood.

In this study, we aimed to identify consistently dysregulated genes in lung adenocarcinoma (LUAD) through integrative transcriptomic analyses and to explore their functional relevance, network centrality, and prognostic significance. We further sought to evaluate whether any candidate hub genes, particularly secreted factors, may serve as potential biomarkers related to metastatic behavior in NSCLC. Based on these analyses, we selected FGF2 for functional validation to investigate its potential role in LUAD cell motility.

## 2. Materials and Methods

### 2.1. Data Collection from Public GEO Datasets

Three gene expression datasets—GSE19804, GSE19188, and GSE18842—were obtained from the publicly available Gene Expression Omnibus (GEO) database (https://www.ncbi.nlm.nih.gov/geo/, accessed on 1 May 2025) for comparative transcriptomic analysis between NSCLC and normal lung tissues [22,23,24]. GSE19804 (last updated: 15 January 2020) includes 60 NSCLC tissue samples and 60 matched adjacent normal lung tissues, based on the GPL570 Affymetrix Human Genome U133 Plus 2.0 Array platform. GSE19188 (last updated: 25 March 2019) contains 65 lung tumor samples and 45 normal lung tissues, also generated using the GPL570 platform. GSE18842 (last updated: 25 March 2019) comprises 46 lung cancer tissues and 45 normal samples, similarly profiled on the GPL570 platform. All datasets were processed using standardized bioinformatics pipelines and analyzed using publicly available platforms to identify differentially expressed genes (DEGs) and explore their relevance to lung cancer progression and metastasis.

### 2.2. Identification of DEGs

Differential expression analysis for the three GEO datasets was performed using the GEO2R platform (R v4.2.2), which is built upon the GEOquery (v2.66.0) and limma Bioconductor packages (v3.54.0) [25]. Briefly, differential expression analysis was performed using GEO2R (GEOquery + limma). GEO2R applies automatic log_2_ transformation when required (“auto-detect” mode) and quantile normalization when indicated by the dataset metadata. Precision weights (voom) and forced normalization were not applied. GEO2R automatically removes exact duplicate probe entries. Probe IDs were mapped to gene symbols based on GPL570 platform annotations. Because multiple probes may map to the same gene, downstream intersection analyses were performed at the Gene.symbol level. DEG computation followed the standard limma workflow used by GEO2R (lmFit, eBayes, and topTable), with Benjamini–Hochberg correction for multiple testing. Genes were considered DEGs if adj.P.Val < 0.01 and |log_2_FC| ≥ 1. This yielded 1404 DEGs (GSE19804), 3194 DEGs (GSE18842), and 1850 DEGs (GSE19188).

InteractiVenn (https://www.interactivenn.net/, accessed on 5 May 2025) was then used to identify overlapping DEGs; during this process, entries lacking valid Gene.symbol annotations were automatically removed, and duplicate gene symbols were collapsed [26]. The final intersected DEG set contained 949 unique genes. As requested, full DEG tables for all datasets—including ID, logFC, SE, raw *p*-value, adj.P.Val (FDR), t, B, Gene.symbol, and Gene.title—are now provided as Supplementary Files S1–S4.

To evaluate the robustness of the intersection-based DEG strategy, a sensitivity analysis was performed by conducting a random-effects meta-analysis of differential expression effect sizes for the 11 hub genes. For each dataset (GSE19804, GSE18842, GSE19188), log_2_ fold changes (log_2_FC) and standard errors were extracted directly from the limma output. Meta-analysis was conducted using a DerSimonian–Laird random-effects model, generating pooled effect sizes with 95% confidence intervals (CI). Forest plots include study-level estimates, weights, pooled log_2_FC, and heterogeneity statistics (I^2^). The full results and forest plots are provided in Supplementary File S5.

### 2.3. GO and KEGG Pathway Enrichment of DEGs

To explore the biological significance of the identified DEGs, we conducted functional enrichment analysis using multiple bioinformatics platforms. The Cancer Hallmarks Analytics Tool (https://cancerhallmarks.com/, accessed on 5 May 2025) was utilized to assess the association of DEGs with key oncogenic processes and hallmark traits of cancer, such as proliferation, angiogenesis, invasion, and immune evasion. For a comprehensive understanding of the underlying cellular functions and signaling pathways, we performed gene set enrichment analysis (GSEA) using ShinyGO (version 0.82; http://bioinformatics.sdstate.edu/go/, accessed on 5 May 2025) [27,28,29]. This platform enabled us to carry out Gene Ontology (GO) and Kyoto Encyclopedia of Genes and Genomes (KEGG) enrichment analyses. GO analysis categorized DEGs into three domains: biological processes (BP), molecular functions (MF), and cellular components (CC), thereby providing insights into their functional roles at the molecular and cellular levels. KEGG analysis was used to identify relevant signaling pathways associated with lung cancer development and metastasis. All enrichment analyses applied stringent statistical thresholds, with a *p*-value < 0.01 and a false discovery rate (FDR) < 0.05 to ensure the reliability of results. Enriched terms were ranked based on FDR values, number of mapped genes, and enrichment scores. These analyses facilitated the interpretation of functional networks and highlighted biological themes relevant to NSCLC progression.

### 2.4. Protein–Protein Interaction (PPI) Network Analysis and Hub Gene Selection

To explore the interactions among the 949 overlapping DEGs, a protein–protein interaction (PPI) network was constructed using STRING (version 12.0; accessed on 15 May 2025; https://string-db.org/) [30,31]. The minimum required interaction score was set to 0.400, corresponding to STRING’s “medium confidence” threshold, which reflects a moderate probability that an interaction is true based on aggregated experimental, computational, and transferred evidence. All other STRING parameters were maintained at default settings. The STRING-derived network was imported and visualized using Cytoscape version 3.10.2, an open-source platform for biological network analysis [32]. To identify highly interconnected regions within the PPI network, we applied the MCODE (Molecular Complex Detection) plugin, version 1.5.1. Clustering was performed using the parameters (Degree Cutoff = 2, Node Score Cutoff = 0.2, K-Core = 2, and Max Depth = 100). MCODE identified multiple subnetworks (clusters). The highest-scoring cluster (Score = 62.646, comprising 66 nodes and 2036 edges) was selected for subsequent hub-gene analysis, as it exhibited the greatest network density and functional connectivity. To identify central regulatory genes within the highest-scoring module, we applied the CytoHubba plugin in Cytoscape [32,33]. Three complementary topological algorithms were used to rank gene importance: Degree (number of direct interactions), Closeness (inverse of total distance to all other nodes), and Maximum Neighborhood Component (MNC). For each algorithm, the top 15 genes were extracted. The intersection of the three ranking lists was used to define the final set of 11 robust hub genes, reducing algorithm-specific bias. Full ranked gene lists from each algorithm are provided in Supplementary File S6, as recommended. The topology-derived “hubness” reflects network centrality only and should be regarded as hypothesis-generating rather than causal evidence. Experimental or mechanistic validation is required to establish biological function. This integrative approach yielded 11 consistently ranked hub genes, including: FOS (FBJ murine osteosarcoma viral oncogene homolog), MMP9 (Matrix metallopeptidase 9), IL6 (Interleukin-6), PTGS2 (Prostaglandin-endoperoxide synthase 2), IL1B (Interleukin-1β), ICAM1 (Intercellular adhesion molecule 1), KDR (Kinase insert domain receptor/VEGFR), CCL2 (C-C motif chemokine ligand 2), PECAM1 (Platelet and endothelial cell adhesion molecule 1), FGF2 (Fibroblast growth factor 2), and TLR4 (Toll-like receptor 4). These hub genes were considered putative key regulators of NSCLC progression and were selected for further downstream analysis. A detailed step-by-step workflow of all downstream analyses performed using web-based and GUI tools is provided in Supplementary File S10.

### 2.5. Prognostic Significance and Expression Validation of Hub Genes

To assess the prognostic significance of the identified hub genes in NSCLC, we utilized the Kaplan–Meier Plotter (https://kmplot.com/analysis/; accessed on 16 May 2025), an online tool that correlates gene expression with patient survival outcomes based on multiple publicly available datasets [34,35]. Survival curves were generated using the auto-selected optimal cutoff, and statistical analysis was performed using Cox proportional hazards regression to evaluate overall survival (OS) and first progression survival (FP) differences between high and low gene expression groups. To avoid significance inflation associated with the Kaplan–Meier-Plotter “auto select best cutoff,” survival analyses were also performed using predefined expression tertiles. For each hub gene, patients were split into T1 (low expression) and T3 (high expression) groups, and univariate Cox proportional hazards models were used to estimate HRs and 95% confidence intervals for overall survival (OS) and first progression (FP). In total, 22 Cox models (11 genes × 2 endpoints) were conducted. Complete numerical results are provided in Supplementary File S7. To enhance the robustness and generalizability of our survival findings, we further employed the Lung Cancer Explorer (LCE) platform (https://lce.biohpc.swmed.edu/lungcancer/; accessed on 20 May 2025). This resource integrates transcriptomic and clinical data from multiple NSCLC cohorts, allowing for comprehensive meta-analysis, cross-study comparisons, and expression correlation analyses across independent datasets. To investigate the expression profiles of candidate hub genes across different tumor stages and metastatic statuses, we analyzed mRNA and protein expression using multiple publicly available bioinformatics resources. The UALCAN portal (https://ualcan.path.uab.edu; accessed on 19 May 2025), which provides access to The Cancer Genome Atlas (TCGA) transcriptomic and proteomic data, was used to evaluate FGF2 expression in relation to clinical stage and lymph node involvement in NSCLC patients [36,37]. For additional protein-level validation, immunohistochemistry (IHC) data were retrieved from the Human Protein Atlas (version 24.0) (https://www.proteinatlas.org/; accessed on 16 May 2025) [38]. FGF2 protein localization and staining intensity were compared between normal lung tissues and LUAD samples to verify its differential expression pattern.

### 2.6. Cell Lines and Culture

The human NSCLC cell lines CL1-0 and CL1-5 were kindly provided by Dr. Pan-Chyr Yang (National Taiwan University, Taiwan). The CL1-0 cell line was originally established from a 64-year-old male patient diagnosed with poorly differentiated lung adenocarcinoma. CL1-5, a more invasive subline, was subsequently derived from CL1-0 through in vitro selection for enhanced migratory capacity. Both cell lines were maintained in RPMI-1640 medium supplemented with 10% fetal bovine serum (FBS) and cultured under standard conditions at 37 °C in a humidified incubator with 5% CO_2_. All cells were routinely tested to ensure the absence of mycoplasma contamination and were used at low passage numbers for experimental consistency. Both CL1-0 and CL1-5 cell lines were authenticated by short tandem repeat (STR) profiling using the GenePrint^®^ 24 System (TRI-I Biotech, Taipei, Taiwan; Report No. CA2511210003; 1 December 2025). The complete STR report is provided in Supplementary File S9. The recombinant human FGF2 used in this study was purchased from PeproTech (Human FGF-basic, 154 aa; catalog #100-18B, Thermo Fisher Scientific, Waltham, MA, USA). A working concentration of 10 ng/mL FGF2 was selected based on prior studies demonstrating that this dose is widely used to elicit biologically relevant FGFR-mediated responses, including enhanced cell migration, morphological changes, and transcriptional regulation across multiple cell types [39,40,41,42,43]. These studies consistently show that 10 ng/mL FGF2 is sufficient to activate downstream signaling without inducing cytotoxicity. In addition, cell viability was assessed by CCK-8 after 24 h exposure to FGF2 (0–10 ng/mL) in both CL1-0 and CL1-5 cells, showing no significant cytotoxicity (Supplementary File S8). Therefore, the same concentration was applied in our functional assays to ensure comparability with established experimental conditions.

### 2.7. Transwell Migration and Wound Healing Assays

To assess cell migratory capacity, CL1-0 and CL1-5 cells (2 × 10^4^ cells/well) were seeded into the upper chambers of 8-μm pore size Transwell inserts (Cat. No. 3428; Costar, New York, NY, USA). After 24 h of incubation, non-migrated cells remaining on the upper surface of the membrane were carefully removed with a cotton swab. Migrated cells on the lower surface were fixed with 4% formaldehyde for 15 min at room temperature and subsequently stained with 0.05% crystal violet for 45 min. Stained cells were imaged and counted under a microscope. Quantification was performed using ImageJ software (version 1.52a; NIH, Bethesda, MD, USA). For the wound healing assay, 2.5 × 10^4^ CL1-0 or CL1-5 cells were plated in two-well Culture-Inserts (Cat. No. 80209; ibidi GmbH, Munich, Germany). After 24 h of culture to achieve cell confluency, the inserts were gently removed to generate a consistent cell-free gap (“wound”). Cells were then incubated in serum-containing medium with or without FGF2 treatment, and wound closure was monitored microscopically at 0 and 24 h. Images were captured at each time point. All Transwell migration experiments were performed using three independent biological replicates, each containing three technical replicate wells. For each well, two randomly selected non-overlapping microscopic fields were imaged and quantified. Wound healing assays were conducted using four independent biological replicates, and measurements were obtained from the same standardized regions across wells, with wound closure quantified using ImageJ. All image acquisition and quantification were performed by an investigator blinded to the experimental treatment groups. The raw quantification spreadsheets for Transwell migration counts and wound-closure measurements are provided in Supplementary File S11.

### 2.8. Statistical Analysis

Statistical analyses of gene expression differences between NSCLC and normal lung tissues from TCGA datasets were conducted using two-tailed unpaired Student’s *t*-tests, with Benjamini–Hochberg correction applied to control for false discovery in multiple comparisons. Kaplan–Meier survival curves and univariate Cox proportional hazards models were employed to assess the prognostic significance of gene expression levels. For experimental assays, comparisons between two groups were analyzed using unpaired two-tailed Student’s *t*-tests. When more than two groups were involved, data were analyzed using two-way analysis of variance (ANOVA) followed by Sidak’s post hoc test for pairwise comparisons. All data are presented as mean ± standard deviation (SD). A *p*-value < 0.05 was considered statistically significant.

## 3. Results

### 3.1. Identification of Common Differentially Expressed Genes in NSCLC

**Figure 1:**
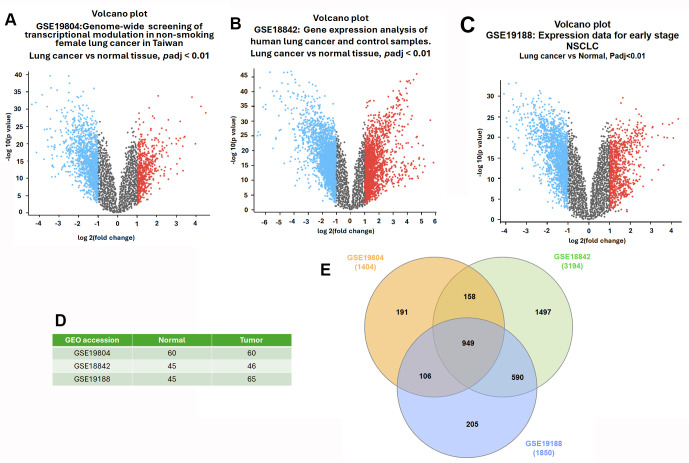
Identification of differentially expressed genes (DEGs) in non-small cell lung cancer (NSCLC) using publicly available GEO datasets. (**A**–**C**) Volcano plots depicting significantly upregulated (red) and downregulated (blue) genes, with non-significant genes shown in gray, in NSCLC tumor tissues compared to normal lung tissues across the three datasets: GSE19804, GSE18842, and GSE19188. DEGs were identified using adjusted *p*-value < 0.01 and |log_2_ fold change| ≥ 1 as cutoffs. (**D**) Summary of sample composition in each dataset: GSE19804 includes 60 tumor and 60 normal samples; GSE18842 includes 46 tumor and 45 normal samples; GSE19188 includes 65 tumor and 45 normal samples. (**E**) Venn diagram illustrating the number of DEGs identified in each dataset and their intersection. The total number of DEGs identified were 1404 (GSE19804), 3194 (GSE18842), and 1850 (GSE19188), with 949 overlapping DEGs common to all three datasets.

To uncover robust and clinically relevant differentially expressed genes (DEGs) associated with non-small cell lung cancer (NSCLC), we analyzed three independent transcriptomic datasets retrieved from the Gene Expression Omnibus (GEO): GSE19804, GSE18842, and GSE19188. Each dataset contains paired or adjacent normal and tumor lung tissue samples, enabling a comparative expression analysis between cancerous and non-cancerous tissues. The sample sizes were as follows: GSE19804 included 60 tumor and 60 normal samples; GSE18842 comprised 46 tumor and 45 normal samples; and GSE19188 consisted of 65 tumor and 45 normal samples (Figure 1D). Using GEO2R, we performed differential expression analysis for each dataset individually, identifying genes significantly upregulated or downregulated in NSCLC tissues compared to normal controls. The resulting DEGs were visualized with volcano plots to illustrate the distribution of significant fold changes and adjusted *p*-values (Figure 1A–C). The number of DEGs identified in each dataset was: 1404 in GSE19804, 3194 in GSE18842, and 1850 in GSE19188. To pinpoint genes consistently dysregulated across all datasets, we conducted an intersection analysis using a Venn diagram, which revealed a total of 949 shared DEGs (Figure 1E). These overlapping genes represent a conserved transcriptional signature in NSCLC and were subsequently selected for downstream enrichment, network, and prognostic analyses due to their potential biological and clinical relevance.

### 3.2. Functional Enrichment Analyses Highlight Metastasis-Associated Hallmarks and Signaling Pathways in LUAD

**Figure 2:**
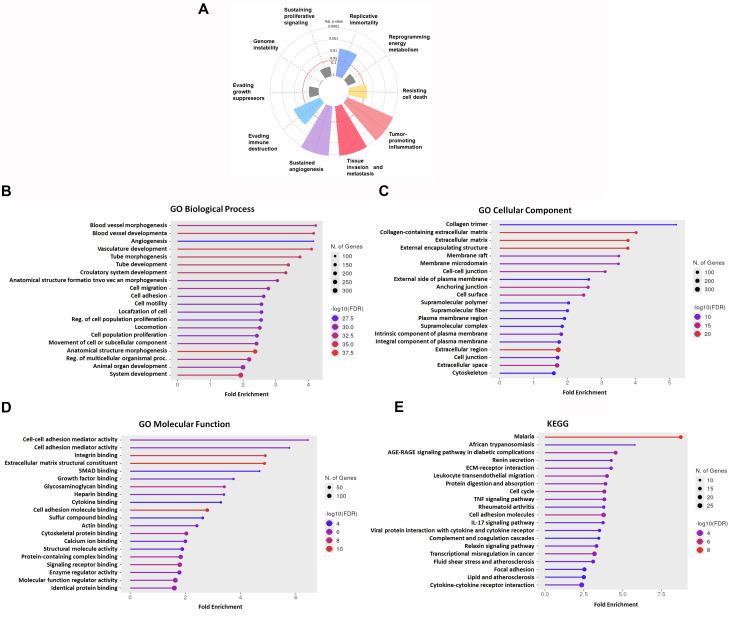
Functional enrichment analysis of shared DEGs in NSCLC. (**A**) Hallmark gene set enrichment analysis of the 949 overlapping differentially expressed genes (DEGs) identified from GSE19804, GSE18842, and GSE19188. *p*-values < 0.05 were considered statistically significant. (**B**) Gene Ontology (GO) Biological Process enrichment analysis. (**C**) GO Cellular Component analysis. (**D**) GO Molecular Function analysis. (**E**) Kyoto Encyclopedia of Genes and Genomes (KEGG) pathway analysis.

To gain deeper insights into the biological functions and clinical implications of the 949 DEGs commonly identified across the three GEO datasets, we first conducted a hallmark gene set enrichment analysis using the Cancer Hallmark Analytics Tool. The results demonstrated that the DEGs were significantly enriched in multiple cancer-related hallmark categories, including evading immune destruction (adjusted *p*-values < 0.01), replicative immortality (adjusted *p*-values < 0.01), resisting cell death (adjusted *p*-values < 0.05), sustained angiogenesis (adjusted *p*-values < 0.001), tumor-promoting inflammation (adjusted *p*-values < 0.001), and tissue invasion and metastasis (adjusted *p*-values < 0.001) (Figure 2A). These results suggest that the shared DEGs are intimately involved in tumor progression processes, particularly those governing angiogenesis, immune escape, and metastatic dissemination in LUAD. To further investigate the biological relevance of the 949 overlapping DEGs, we performed functional enrichment analysis using Gene Ontology (GO) and Kyoto Encyclopedia of Genes and Genomes (KEGG) databases. GO Biological Process (BP) analysis revealed that DEGs were significantly enriched in pathways related to angiogenesis, vasculature development, cell migration, cell adhesion, and multicellular organismal development—processes closely linked to tumor progression and metastasis (Figure 2B). In the Cellular Component (CC) category, DEGs were predominantly localized to the extracellular matrix, cell–cell junctions, plasma membrane, and cytoskeleton, suggesting active involvement in remodeling of the tumor microenvironment (Figure 2C). Molecular Function (MF) analysis highlighted significant enrichment in integrin binding, extracellular matrix structural components, growth factor and cytokine binding, and actin-binding activity, reinforcing the role of these genes in cell motility, immune signaling, and cell–matrix interactions (Figure 2D). Consistent with GO results, KEGG pathway analysis revealed enrichment in several cancer- and metastasis-related pathways, including ECM–receptor interaction, focal adhesion, cytokine–cytokine receptor interaction, IL-17 signaling, leukocyte transendothelial migration, and TNF signaling (Figure 2E). These pathways are well-recognized for their roles in tumor invasion, immune evasion, and vascular dissemination. Collectively, these enrichment results suggest that the core DEGs identified in LUAD are strongly associated with key mechanisms of metastasis and tumor aggressiveness, highlighting their potential utility as prognostic biomarkers and therapeutic targets.

### 3.3. Identification and Expression Profiling of Hub Genes in NSCLC

**Figure 3:**
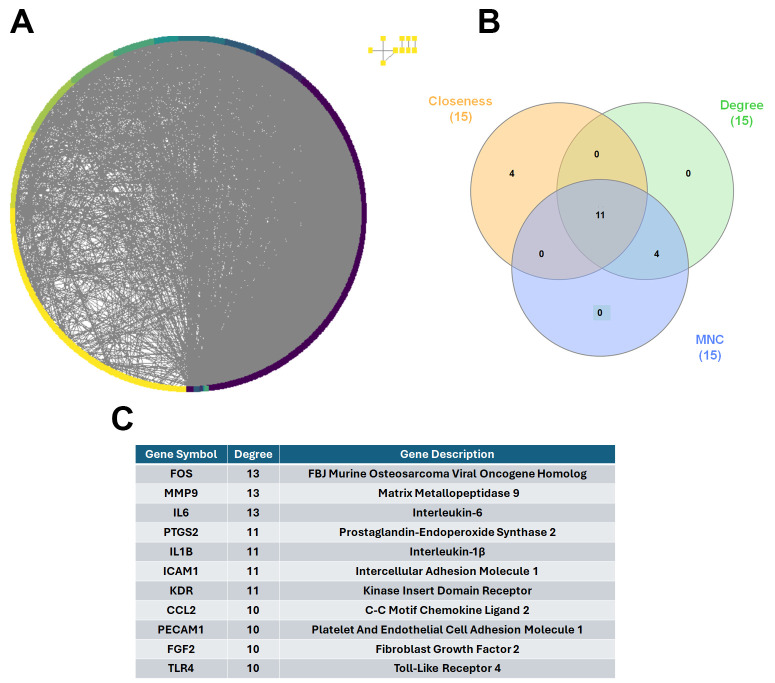
Identification of hub genes in NSCLC through PPI network analysis. (**A**) Protein–protein interaction (PPI) network constructed using 949 overlapping differentially expressed genes (DEGs) from GSE19804, GSE18842, and GSE19188, based on STRING database analysis. (**B**) Venn diagram displaying the intersection of the top 15 hub genes identified by three network topology algorithms—Closeness, Degree, and Maximum Neighborhood Component (MNC)—using the CytoHubba plugin in Cytoscape. (**C**) Final list of 11 hub genes consistently ranked across all three algorithms: FOS, MMP9, IL6, PTGS2, IL1B, ICAM1, KDR, CCL2, PECAM1, FGF2, and TLR4.

Building upon the enrichment analyses that linked the identified DEGs to metastasis-related processes, we sought to uncover key regulatory genes that may drive NSCLC progression. To this end, a protein–protein interaction (PPI) network was constructed using the 949 overlapping DEGs via the STRING database (https://string-db.org/) (accessed on 15 May 2025). The resulting interaction network was imported into Cytoscape for visualization and modular analysis (Figure 3A). Using the CytoHubba plugin within Cytoscape, we applied three established topological algorithms—Closeness centrality, Degree, and Maximum Neighborhood Component (MNC)—to rank genes based on their centrality within the network. The top 15 genes identified by each algorithm were compared, and a Venn diagram was used to determine the intersection. This approach yielded 11 consistently ranked hub genes (Figure 3B), including: FOS, MMP9, IL6, PTGS2, IL1B, ICAM1, KDR, CCL2, PECAM1, FGF2, and TLR4 (Figure 3C).

**Figure 4:**
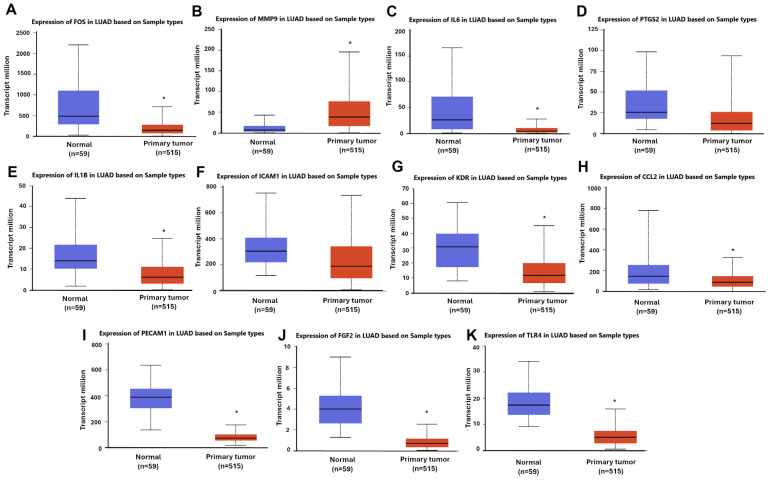
Transcript expression levels of hub genes in LUAD based on TCGA data. Relative mRNA expression levels of the 11 hub genes in LUAD (*n* = 515) versus normal lung tissues (*n* = 59), analyzed using the UALCAN web portal. (**A**–**K**) represent the expression levels of FOS (**A**), MMP9 (**B**), IL6 (**C**), PTGS2 (**D**), IL1B (**E**), ICAM1 (**F**), KDR (**G**), CCL2 (**H**), PECAM1 (**I**), FGF2 (**J**), and TLR4 (**K**) in normal and LUAD tissues. Statistically significant differences between groups are indicated by an *, corresponding to *p*-values < 0.05.

To evaluate the clinical relevance of these hub genes, we analyzed their transcript expression levels in LUAD tissues versus normal lung tissues using the UALCAN platform, which sources data from the TCGA database (Figure 4). Among the 11 hub genes, FOS, IL6, IL1B, KDR, CCL2, PECAM1, FGF2, and TLR4 were significantly downregulated in LUAD samples compared to normal controls. In contrast, MMP9 exhibited significantly elevated expression in LUAD tissues. Although PTGS2 and ICAM1 displayed slightly lower median expression levels in LUAD compared with normal tissues in the TCGA-derived boxplots, both genes showed substantial intragroup variability, and the corresponding *t*-tests did not reach statistical significance. Thus, while the expression patterns appear altered, the statistical support for these differences remains weak. These expression trends suggest that many of the identified hub genes may represent potential tumor suppressors or pro-metastatic drivers in NSCLC. Their dysregulation underscores their possible involvement in LUAD progression and warrants further functional validation.

### 3.4. Prognostic Value of Hub Genes in LUAD Patients

**Figure 5:**
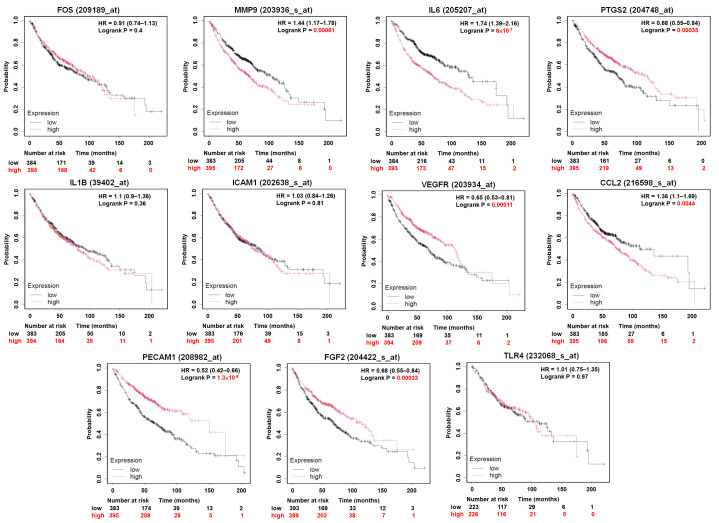
Overall survival (OS) analysis of hub genes in LUAD patients. Kaplan–Meier survival curves depicting the association between mRNA expression levels of 11 hub genes and overall survival (OS) in patients with LUAD, based on TCGA data analyzed using the Kaplan–Meier Plotter platform. Log-rank *p*-values < 0.05 were considered statistically significant.

**Figure 6:**
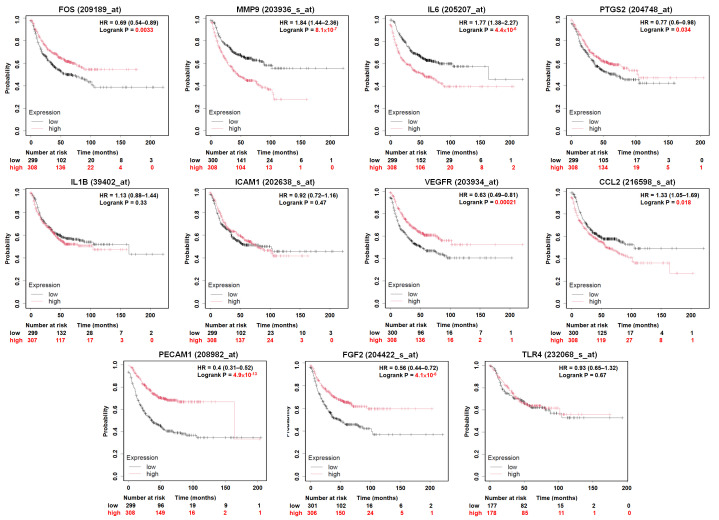
First progression survival (FP) analysis of hub genes in LUAD patients. Kaplan–Meier curves showing first progression survival (FP) stratified by high and low expression of the 11 hub genes in LUAD, using data from the Kaplan–Meier Plotter. Log-rank *p*-values < 0.05 were considered statistically significant.

To assess the clinical relevance of the identified hub genes, we evaluated their association with patient prognosis in LUAD using the Kaplan–Meier Plotter platform (https://kmplot.com/analysis/, accessed on 16 May 2025). Survival analysis was conducted based on mRNA expression levels, stratifying patients into high and low expression groups using predefined expression tertiles (T1 vs. T3). For overall survival (OS), elevated expression of several genes showed statistically significant associations with patient outcomes. Specifically: High MMP9 expression was associated with significantly shorter survival (log-rank *p* = 0.00051); high IL6 expression correlated with shorter survival (*p* = 6.2 × 10^−9^); high CCL2 expression was also linked to poorer prognosis (*p* = 7.7 × 10^−5^); in contrast, high PTGS2 (*p* = 0.00028), KDR (VEGFR) (*p* = 4.0 × 10^−6^), PECAM1 (*p* = 5.6 × 10^−10^), and FGF2 (*p* = 0.0003) expression were significantly associated with longer overall survival. Expression of the remaining hub genes showed no statistically significant impact on OS (Figure 5). We further evaluated the effect of hub gene expression on first progression survival (FP). The analysis revealed consistent trends with OS: High MMP9, IL6, and CCL2 expression were significantly associated with shorter FP (*p* = 1.7 × 10^−7^, 7.9 × 10^−8^, and 0.001, respectively); conversely, high expression of FOS (*p* = 0.00047), PTGS2 (*p* = 0.021), KDR (*p* = 4.3 × 10^−5^), PECAM1 (*p* = 2.9 × 10^−11^), and FGF2 (*p* = 2.2 × 10^−5^) were significantly associated with prolonged FP. Other genes did not show a significant prognostic impact on FP (Figure 6). Taken together, these results highlight a subset of hub genes whose expression correlates consistently with favorable or unfavorable prognosis in LUAD. Among them, FGF2 stands out as it is not only downregulated in tumor tissues, but its higher expression is also associated with improved survival in both OS and FP analyses. Given its secretory nature and consistent prognostic pattern, FGF2 may serve as a promising candidate for non-invasive disease monitoring or plasma-based metastasis risk assessment in NSCLC.

### 3.5. FGF2 Downregulation Is Associated with Tumor Progression and Reduced Cell Motility in NSCLC

**Figure 7:**
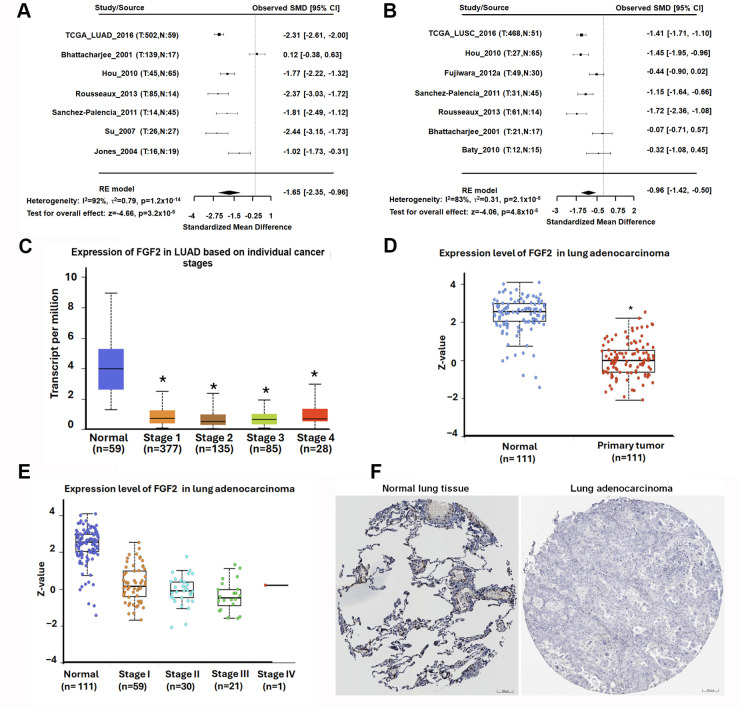
FGF2 is downregulated in NSCLC and associated with advanced tumor stages. (**A**,**B**) Meta-analysis of multiple transcriptomic datasets using the Lung Cancer Explorer platform. (**C**) Analysis of TCGA LUAD data in advanced-stage tumors. (**D**) CPTAC proteomic data of FGF2 protein levels in LUAD tissues versus normal lung tissues. (**E**) Stage-wise comparison from CPTAC of FGF2 protein expression across different LUAD pathological stages. (**F**) IHC staining from the Human Protein Atlas of FGF2 protein expression in LUAD tissues compared to normal lung samples. Scale bar = 100 µm. Statistically significant differences between groups are indicated by an *, corresponding to *p*-values < 0.05.

Following the identification of FGF2 as a hub gene with favorable prognostic significance in NSCLC, we further investigated its role in tumor progression and metastasis. To validate the differential expression of FGF2, we performed an integrative analysis using multiple public datasets and analytical platforms. A meta-analysis conducted through the Lung Cancer Explorer (LCE) platform (https://lce.biohpc.swmed.edu/lungcancer/, accessed on 20 May 2025) revealed that FGF2 mRNA expression was significantly lower in both LUAD (SMD = −1.65) and lung squamous cell carcinoma (LUSC) (SMD = −0.96) tissues compared to normal lung tissues (Figure 7A,B). Consistent with these findings, analysis of TCGA LUAD data demonstrated that FGF2 transcript levels were significantly downregulated in advanced-stage tumors relative to normal controls (Figure 7C). At the protein level, data from the Clinical Proteomic Tumor Analysis Consortium (CPTAC) showed a marked reduction in FGF2 protein expression in LUAD tumor samples (Figure 7D), and this downregulation remained consistent across different pathological stages (Figure 7E). Furthermore, IHC staining data from the Human Protein Atlas (https://www.proteinatlas.org/ENSG00000138685-FGF2/cancer, accessed on 16 May 2025) confirmed weak FGF2 staining in LUAD tissues, further supporting transcriptomic and proteomic evidence (Figure 7F).

**Figure 8:**
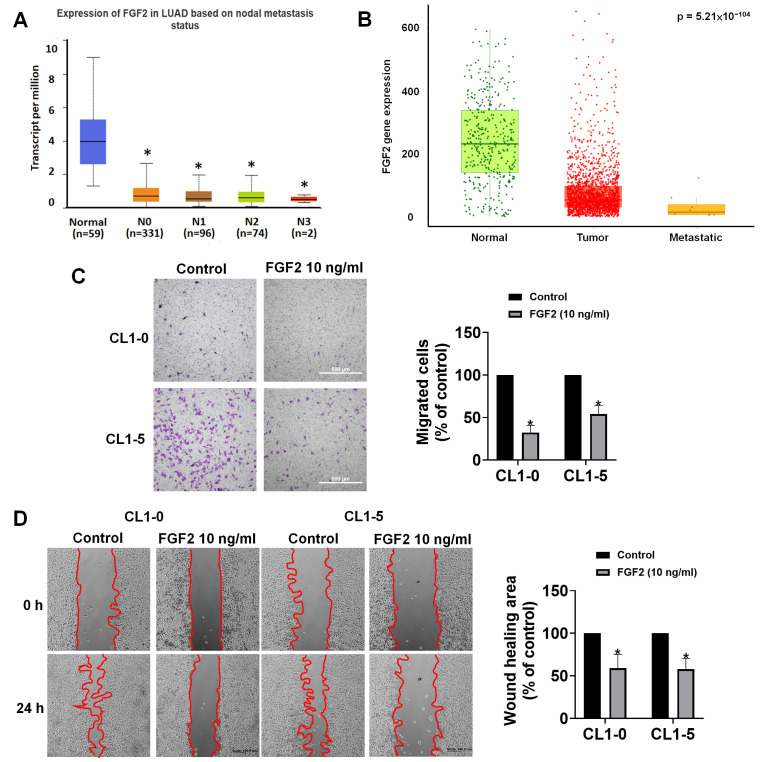
FGF2 is downregulated in metastatic NSCLC and exogenous FGF2 treatment reduces lung cancer cell motility in vitro. (**A**) Expression analysis of FGF2 mRNA levels across lymph node metastasis stages (N0–N3) in LUAD using TCGA data. (**B**) Comparison of FGF2 expression in normal tissues, primary tumors, and metastatic lesions based on gene chip datasets from the TNMplot platform. (Kruskal–Wallis *p* = 5.78 × 10^−104^). (**C**) Transwell migration assay of CL1-0 and CL1-5 lung cancer cells treated with FGF2 (10 ng/mL) for 24 h. (*n* = 3) Scale bar: 500 μm (**D**) Wound healing assay further confirmed the effect of FGF2 on the wound closure ability of both CL1-0 and CL1-5 cells. (*n* = 4) Scale bar: 100 μm Each assay represents at least three biological replicates, with two fields per well quantified by a blinded evaluator. Data represent mean ± SD. Statistically significant differences between groups are indicated by an *, corresponding to *p*-values < 0.05.

To determine whether FGF2 expression is related to metastatic behavior, we examined its expression in metastatic lymph node tissues using TCGA data. Results indicated that FGF2 transcript levels decreased progressively with increasing lymph node metastasis (Figure 8A). To further assess expression across disease states, we utilized the TNMplot platform (https://tnmplot.com/analysis/, accessed on 22 May 2025) to compare FGF2 expression in normal tissues, primary tumors, and metastatic lesions based on gene chip datasets. Kruskal–Wallis analysis revealed a significant reduction in FGF2 expression across these tissue types (*p* = 5.78 × 10^−104^), with the lowest expression observed in metastatic tumors (Figure 8B). To experimentally assess the role of FGF2 in NSCLC metastasis, we performed in vitro functional assays using CL1-0 and CL1-5 NSCLC cell lines. In Transwell migration assay, treatment with FGF2 (10 ng/mL) for 24 h significantly inhibited cell migration in both CL1-0 and CL1-5 cells (Figure 8C). Similarly, wound healing assay demonstrated that FGF2 treatment markedly impaired the ability of both cell lines to close the wound gap, indicating suppressed motility (Figure 8D). Together, these findings from multi-omics databases and in vitro experiments consistently indicate that FGF2 is downregulated during NSCLC progression and are consistent with an inhibitory effect of exogenous FGF2 on cancer cell motility in vitro. Given its secreted nature and reduced levels in metastatic tissues, FGF2 represents a plausible candidate for further evaluation as a non-invasive biomarker for NSCLC metastasis in plasma-based screening approaches.

## 4. Discussion

Lung cancer remains the leading cause of cancer-related mortality worldwide, with non-small cell lung cancer (NSCLC) accounting for approximately 85% of all cases. The poor prognosis of NSCLC is largely attributable to late-stage diagnosis and frequent distant metastases at initial presentation. While significant advances have been made in surgical techniques, chemotherapy, targeted therapy, and immunotherapy, many patients still experience therapeutic resistance or lack of response, highlighting the urgent need for novel prognostic biomarkers and therapeutic targets [44,45]. Fibroblast growth factor 2 (FGF2) has been implicated in multiple physiological and pathological processes, including embryonic development, tissue repair, angiogenesis, and cancer. In various malignancies, FGF2 is known to enhance tumor progression by promoting cell proliferation, neovascularization, and metastatic spread [15,46]. However, its role in lung cancer—particularly NSCLC—remains controversial and context-dependent. While some studies have demonstrated a pro-tumorigenic role for FGF2, others have reported inhibitory effects on cell growth and invasion, suggesting dual and possibly tissue-specific functions [16,17,18].

In this study, we performed a comprehensive bioinformatics analysis using three independent GEO datasets and identified 949 consistently dysregulated DEGs in LUAD. GO and KEGG enrichment analyses showed that many of these genes, including FGF2, were associated with cell adhesion, extracellular matrix interactions, and migration-related pathways, which are critical processes in metastasis. Through protein–protein interaction (PPI) network analysis, FGF2 emerged as one of the 11 core hub genes with prognostic significance. Public database analysis consistently revealed downregulation of FGF2 at both the mRNA and protein levels in LUAD and LUSC samples compared to normal tissues, including in advanced and metastatic stages. This trend was further confirmed using CPTAC proteomics and IHC data from the Human Protein Atlas. Moreover, survival analysis indicated that higher FGF2 expression was associated with longer overall and progression-free survival, consistent with a potential tumor-suppressive role in NSCLC. Notably, our in vitro assays demonstrated that FGF2 treatment significantly reduced the migratory capacity of both low-invasive (CL1-0) and high-invasive (CL1-5) lung cancer cell lines. These findings suggest that FGF2 may negatively regulate tumor cell motility, possibly by modulating cell adhesion-related pathways.

Interestingly, the regulatory role of FGF2 varies across cancer types. In breast and prostate cancers, FGF2 has been shown to promote tumor growth and resistance mechanisms—such as driving estrogen receptor signaling in endocrine-resistant breast cancer and enhancing glycolysis via LDHA activation in prostate cancer [47,48]. It also contributes to cancer stem cell maintenance in colorectal cancer and invasion in pancreatic ductal adenocarcinoma, where blockade of FGF2 or its receptors significantly reduces cell proliferation and invasiveness [49,50]. Conversely, in specific molecular contexts, FGF2 exhibits anti-proliferative effects. For instance, in FGFR1-amplified breast cancer, FGF2 suppresses growth by upregulating p21 through JAK/STAT signaling, leading to G1/S arrest [17]. In KRas–mutant models, FGF2 induces cellular senescence via epigenetic regulation and aberrant rRNA processing [16,51]. Furthermore, down-regulation of FGF2 leads to increased gastric cancer cell proliferation and invasion via the PI3K/Akt pathway [18]. This dualistic nature highlights the context-dependent complexity of FGF2 signaling, which may be shaped by receptor subtypes, oncogenic mutations, and the tumor microenvironment.

Our study contributes new evidence consistent with an anti-metastatic role of FGF2 in LUAD and provides a novel perspective on this otherwise paradoxical molecule. However, several limitations should be noted. First, our findings are primarily derived from transcriptomic and proteomic datasets and require validation in larger clinical cohorts. Second, while in vitro results support a functional role for FGF2 in suppressing cell migration, the in vivo dynamics of FGF2 within the tumor microenvironment remain unclear. It is also important to consider the complexity of FGF2 isoforms and receptor subtypes, which may exert distinct biological effects. To further contextualize why FGF2 may demonstrate tumor-suppressive associations specifically in LUAD, several biological mechanisms merit consideration. First, LUAD is enriched for KRAS-driven tumors, and recent multi-omics analyses have shown that FGF2 can paradoxically induce oncogene-induced senescence in Ras-amplified cells by activating p21, promoting chromatin remodeling, and impairing cell-cycle progression [16]. This suggests that in a KRAS-mutant background—one of the most common molecular contexts in LUAD—FGF2 may preferentially trigger cytostatic rather than proliferative pathways. Second, LUAD exhibits distinctive patterns of FGFR genetic alterations, fusions, and splice isoforms [21], which can fundamentally alter ligand–receptor coupling strength and downstream signaling balance. Such alterations may shift FGF2 signaling away from its classical pro-angiogenic outputs toward pathways regulating adhesion, differentiation, or motility. Third, single-cell transcriptomic studies have revealed pronounced spatial heterogeneity in the LUAD tumor microenvironment, including region-specific activation of FGFR and TGF-β pathways and differential immune-cell and stromal composition [52]. These microenvironmental cues may further modulate how LUAD cells interpret FGF2 stimulation. Together, these context-specific mechanisms provide biologically plausible explanations for the observed anti-motility phenotype in vitro and the association between higher endogenous FGF2 expression and improved clinical outcomes in LUAD patients.

Finally, although our in vitro assays demonstrate a clear anti-motility effect of exogenous FGF2, the current study did not determine whether this phenotype is mediated through canonical FGFR signaling. FGF2 can activate multiple FGFR isoforms as well as non-canonical pathways, and dissecting these downstream mechanisms requires pharmacologic or genetic perturbation of FGFR activity. As this was beyond the scope of the present work, we have now highlighted this point as a limitation. Future studies incorporating FGFR inhibitors, ligand-blocking antibodies, or pathway-specific reporters will be necessary to clarify whether the suppressive effects of FGF2 on LUAD cell motility are FGFR-dependent or involve alternative signaling routes. In this study, we focused on wound healing and Transwell migration assay as our primary functional readouts, rather than Matrigel-based invasion. This decision was based on two considerations. First, our transcriptomic analyses identified FGF2 as a gene associated predominantly with cell adhesion, cytoskeletal remodeling, and motility-related pathways, rather than with extracellular matrix (ECM) degradation programs, suggesting that migration would be a more direct and biologically relevant phenotype to assess. Second, migration assays provide a sensitive and reproducible measure of early motility changes without the added complexity of Matrigel, whose batch variability can introduce additional confounders unrelated to FGF2 signaling. While invasion assays would further clarify whether FGF2 influences matrix-penetrating behavior, such experiments were beyond the scope of the current study. We have now noted this in our revised manuscript, and future work incorporating Matrigel invasion or 3D ECM-based assays will help determine whether the anti-motility effect of FGF2 extends to bona fide invasive phenotypes.

## 5. Conclusions

This study identifies FGF2 as a consistently downregulated gene in LUAD, with low expression associated with advanced stage, lymph node metastasis, and poorer clinical outcomes. Functional enrichment and cell-based assays are consistent with FGF2 reducing lung cancer cell migration in vitro, potentially through effects on adhesion-related pathways. These findings support the hypothesis that FGF2 may act as a potential tumor suppressor-like factor and prognostic biomarker candidate in LUAD. Given that FGF2 is a secreted factor, it may serve as a promising non-invasive plasma biomarker for assessing metastatic risk. Further research is needed to elucidate the mechanistic functions of FGF2 in lung cancer and to determine its clinical value for disease monitoring and as a potential target for therapeutic intervention.

## Data Availability

The data presented in this study is available on request from the corresponding author.

## Supplementary Materials

The following supporting information can be downloaded at: https://www.mdpi.com/article/10.3390/diagnostics16020250/s1, File S1: List of differentially expressed genes (DEGs) identified from the GSE19804 dataset. DEGs were filtered using an adjusted *p*-value < 0.01 and |log_2_ fold change| ≥ 1. File S2: List of differentially expressed genes (DEGs) identified from the GSE18842 dataset. DEGs were filtered using an adjusted *p*-value < 0.01 and |log_2_ fold change| ≥ 1. File S3: List of differentially expressed genes (DEGs) identified from the GSE19188 dataset. DEGs were filtered using an adjusted *p*-value < 0.01 and |log_2_ fold change| ≥ 1. File S4: Results of Venn diagram analysis showing all possible intersection combinations of DEGs among the three GEO datasets (GSE19804, GSE18842, and GSE19188). File S5: Random-effects meta-analysis of differential expression effect sizes for the 11 hub genes. For each gene, forest plots display dataset-specific log_2_ fold changes (log_2_FC), standard errors, 95% confidence intervals, study weights, heterogeneity statistics (I^2^), and pooled log_2_FC estimates derived from the random-effects model. File S6: Top 15 hub genes identified using three CytoHubba algorithms (Degree, Closeness, and Maximum Neighborhood Component [MNC]) based on the protein–protein interaction network constructed in Cytoscape. File S7: Survival analysis of hub genes in LUAD patients. To avoid significance inflation associated with the KM-Plotter “auto select best cutoff,” predefined expression tertiles were applied. For each hub gene, patients were divided into T1 (low expression) and T3 (high expression) groups, and univariate Cox proportional hazards models were used to estimate hazard ratios (HRs) and 95% confidence intervals for overall survival (OS) and first progression (FP). File S8: Raw optical density (OD) values and quantified cell viability results from CCK-8 assays performed on CL1-0 and CL1-5 cells treated with FGF2 (0, 0.5, 1, 5, and 10 ng/mL) for 24 h. File S9: Short tandem repeat (STR) profiling report for CL1-0 and CL1-5 cell lines, used for cell line authentication. File S10: Analysis workflow description documenting all downstream analyses performed using web-based and GUI tools, including: (1) DEG input, (2) functional enrichment analysis (GO/KEGG) using ShinyGO v0.82, (3) hallmark enrichment analysis using the Cancer Hallmarks Analytics Tool, (4) protein–protein interaction network construction using STRING v12.0, (5) network visualization and module detection using Cytoscape v3.10.2 with the MCODE plugin, and (6) hub-gene prioritization using the CytoHubba plugin. File S11: Raw numerical data and quantified results for Transwell migration and wound-healing assays. The file includes original measurements (cell migration counts/quantification and wound closure values at 0 and 24 h) from CL1-0 and CL1-5 cells under the experimental conditions described in the main text.

## Abbreviations

The following abbreviations are used in this manuscript:

FGF2	Fibroblast growth factor 2
bFGF	Basic fibroblast growth factor
NSCLC	Non-small cell lung cancer
LUAD	Lung adenocarcinoma
LUSC	Lung squamous cell carcinoma
SCLC	Small cell lung cancer
DEGs	Differentially expressed genes
GEO	Gene expression omnibus
GO	Gene ontology
KEGG	Kyoto encyclopedia of genes and genomes
PPI	Protein–protein interaction
BP	Biological process
MF	Molecular function
CC	Cellular component
FDR	False discovery rate
OS	Overall survival
FP	First progression
TCGA	The cancer genome atlas
CPTAC	Clinical proteomic tumor analysis consortium
LCE	Lung cancer explorer
MCODE	Molecular complex detection
MNC	Maximum neighborhood component
VEGFR	Vascular endothelial growth factor receptor
FGFR	Fibroblast growth factor receptor
TNF	Tumor necrosis factor
IL	Interleukin
ICAM1	Intercellular adhesion molecule 1
KDR	Kinase insert domain receptor
PECAM1	Platelet and endothelial cell adhesion molecule 1
CCL2	C-C motif chemokine ligand 2
PTGS2	Prostaglandin-endoperoxide synthase 2
TLR4	Toll-like receptor 4
MAPK	Mitogen-activated protein kinase
PI3K/Akt	Phosphatidylinositol 3-kinase–protein kinase B pathway
GSEA	Gene set enrichment analysis
UALCAN	University of Alabama at Birmingham cancer data portal
TNMplot	Tumor–node–metastasis plotting platform
