# Supplementary material for: FGF2 as a Potential Tumor Suppressor in Lung Adenocarcinoma
Source: Diagnostics (Basel). 2026 Jan 13;16(2):250. doi: 10.3390/diagnostics16020250 (PMC12839716; doi:10.3390/diagnostics16020250)
Supplement: Supplementary file 1 [file diagnostics-16-00250-s001.zip › Supplementary Files S10.pdf]

Analysis Workflow Description (No Code Scripts Required)

Because all downstream analyses were performed using established web-based or graphical-user-interface (GUI) tools, no executable scripts or computational notebooks were generated. Instead, the complete workflow and parameter settings are documented below to ensure reproducibility.

1. DEG Input

The intersected set of 949 DEGs obtained from the Venn diagram comparison was exported as a plain text file and used as the input list for subsequent enrichment and network analyses.

2. Functional Enrichment (GO/KEGG) – ShinyGO v0.82

Website: http://bioinformatics.sdstate.edu/go/

Species: Homo sapiens

Input: 949 gene symbols

Default statistical settings were used (FDR < 0.05).

Outputs: GO BP/CC/MF enrichment tables and KEGG pathway enrichment tables (exported as CSV/PDF).

3. Hallmark Enrichment – Cancer Hallmarks Analytics Tool

Website: https://cancerhallmarks.com/

Input: Same DEG list

Default settings; hallmark categories automatically generated.

4. PPI Network Construction – STRING v12.0

Website: https://string-db.org/

Input: 949 DEGs

Minimum required interaction score: 0.400 (“medium confidence”)

Other parameters: default settings

Network exported as TSV for Cytoscape.

5. Network Visualization and Module Detection – Cytoscape v3.10.2 + MCODE plugin

MCODE settings:

Degree Cutoff = 2

Node Score Cutoff = 0.2

K-Core = 2

Max Depth = 100

Highest-scoring cluster exported for hub-gene analysis.

6. Hub-Gene Prioritization – CytoHubba plugin

Algorithms used: Degree, Closeness, Maximum Neighborhood Component (MNC)

Top 15 genes from each method extracted

Intersection of the three lists yielded 11 hub genes.

No custom scripts were used in this study. All analyses relied on platform-based algorithms, and the complete parameter settings are documented here for reproducibility.
