# Supplementary material for: FGF2 as a Potential Tumor Suppressor in Lung Adenocarcinoma
Source: Diagnostics (Basel). 2026 Jan 13;16(2):250. doi: 10.3390/diagnostics16020250 (PMC12839716; doi:10.3390/diagnostics16020250)
Supplement: Supplementary file 1 [file diagnostics-16-00250-s001.zip › Supplementary Files S5.pdf]

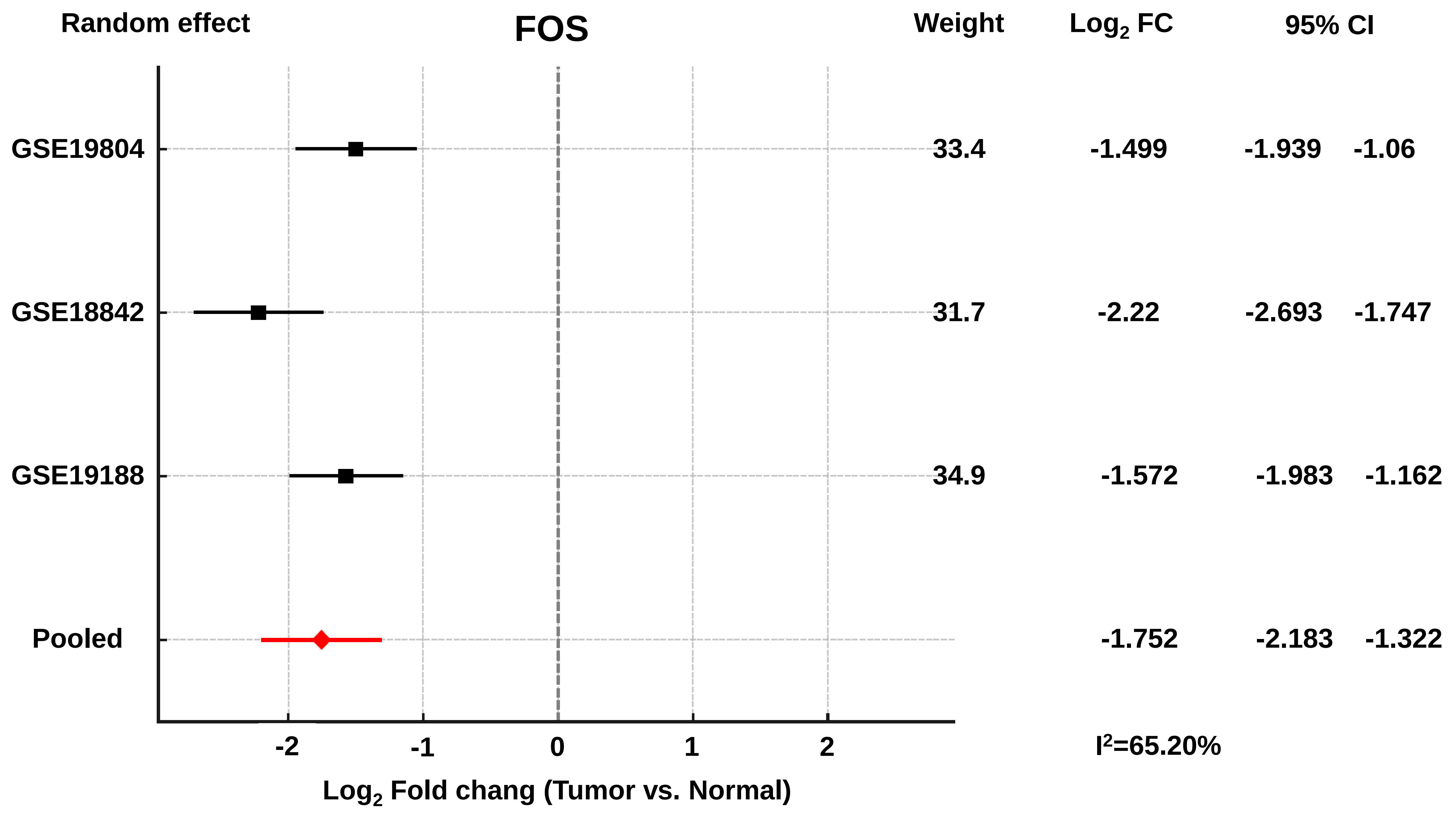
Random effect
FOS
Weight
Log2 FC
95% CI
GSE19804
33.4
-1.499
-1.939
-1.06
GSE18842
31.7
-2.22
-2.693
-1.747
GSE19188
34.9
-1.572
-1.983
-1.162
Pooled
-1.752
-2.183
-1.322
-2
-1
0
1
2
I2=65.20%
Log2 Fold chang (Tumor vs. Normal)

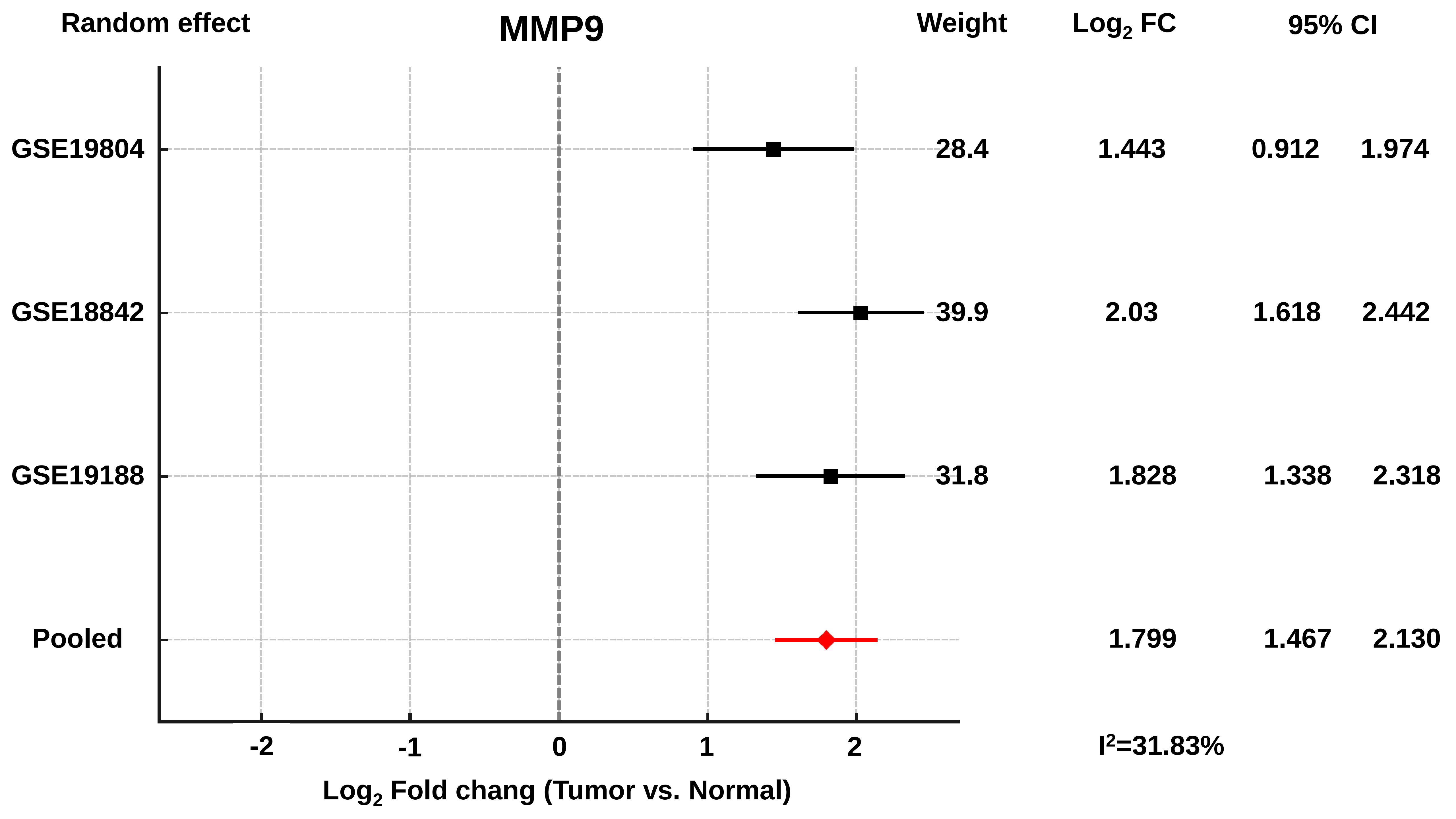
Random effect
MMP9
Weight
Log2 FC
95% CI
GSE19804
28.4
1.443
0.912
1.974
GSE18842
39.9
2.03
1.618
2.442
GSE19188
31.8
1.828
1.338
2.318
Pooled
1.799
1.467
2.130
-2
-1
0
1
2
I2=31.83%
Log2 Fold chang (Tumor vs. Normal)

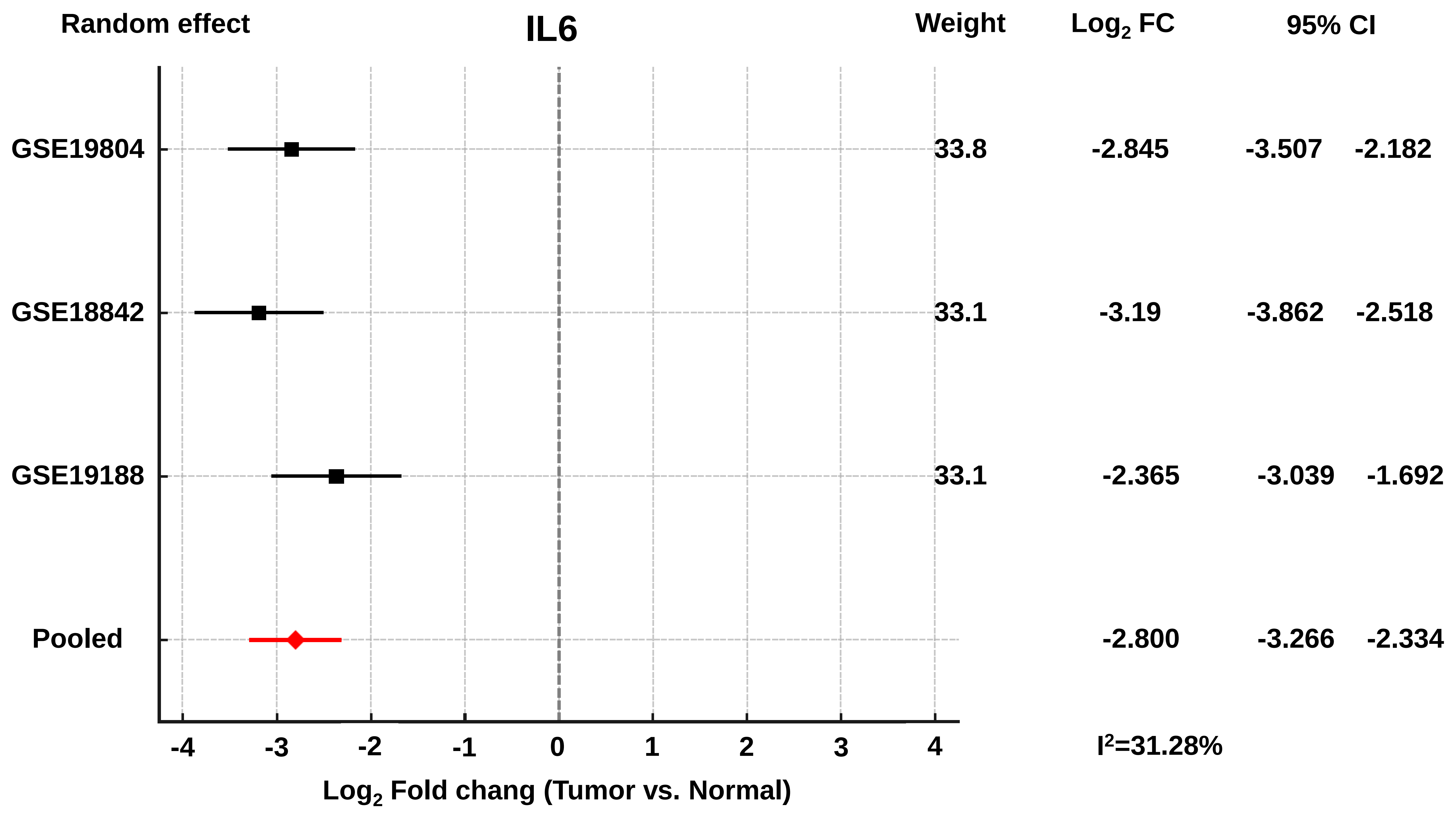
Random effect
IL6
Weight
Log2 FC
95% CI
GSE19804
33.8
-2.845
-3.507
-2.182
GSE18842
33.1
-3.19
-3.862
-2.518
GSE19188
33.1
-2.365
-3.039
-1.692
Pooled
-2.800
-3.266
-2.334
-4
-3
-2
-1
0
1
2
3
4
I2=31.28%
Log2 Fold chang (Tumor vs. Normal)

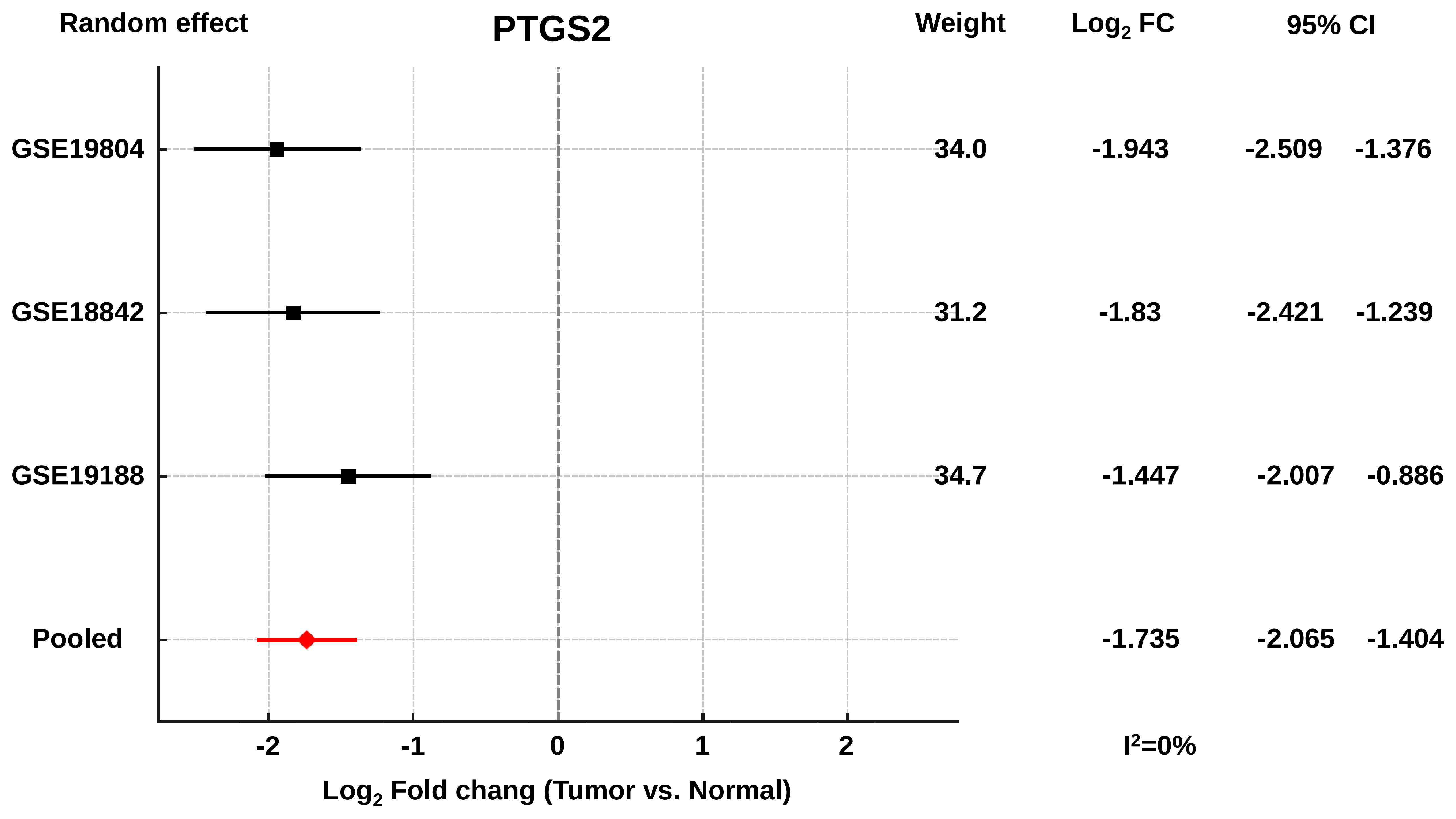

Random effect
PTGS2
Weight
Log2 FC
95% CI
GSE19804
34.0
-1.943
-2.509
-1.376
GSE18842
31.2
-1.83
-2.421
-1.239
GSE19188
34.7
-1.447
-2.007
-0.886
Pooled
-1.735
-2.065
-1.404
-2
-1
0
1
2
I2=0%
Log2 Fold chang (Tumor vs. Normal)

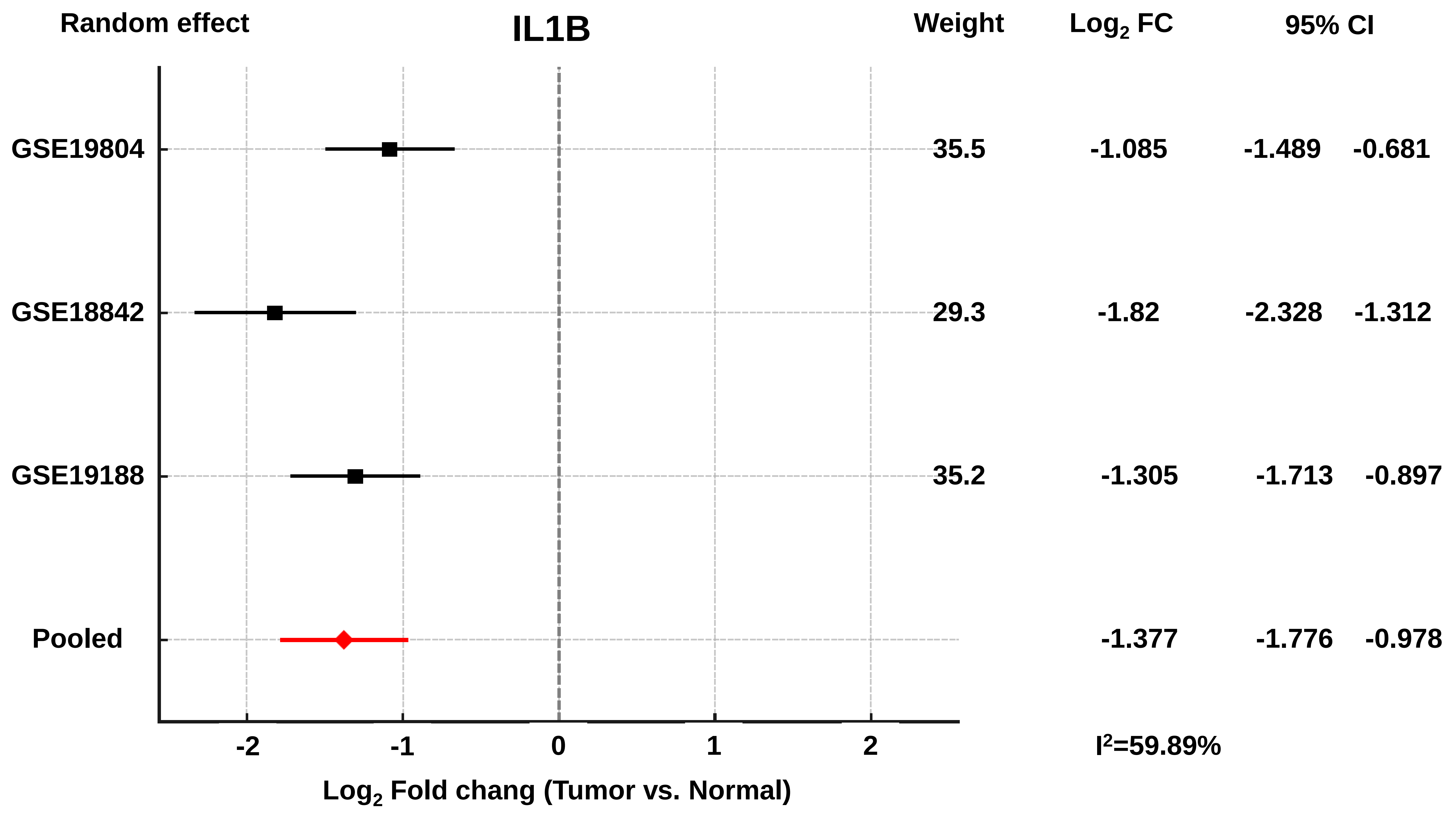
Random effect
IL1B
Weight
Log_2 FC
95% CI
GSE19804
35.5
-1.085
-1.489
-0.681
GSE18842
29.3
-1.82
-2.328
-1.312
GSE19188
35.2
-1.305
-1.713
-0.897
Pooled
-1.377
-1.776
-0.978
-2
-1
0
1
2
I^2=59.89%
Log_2 Fold chang (Tumor vs. Normal)

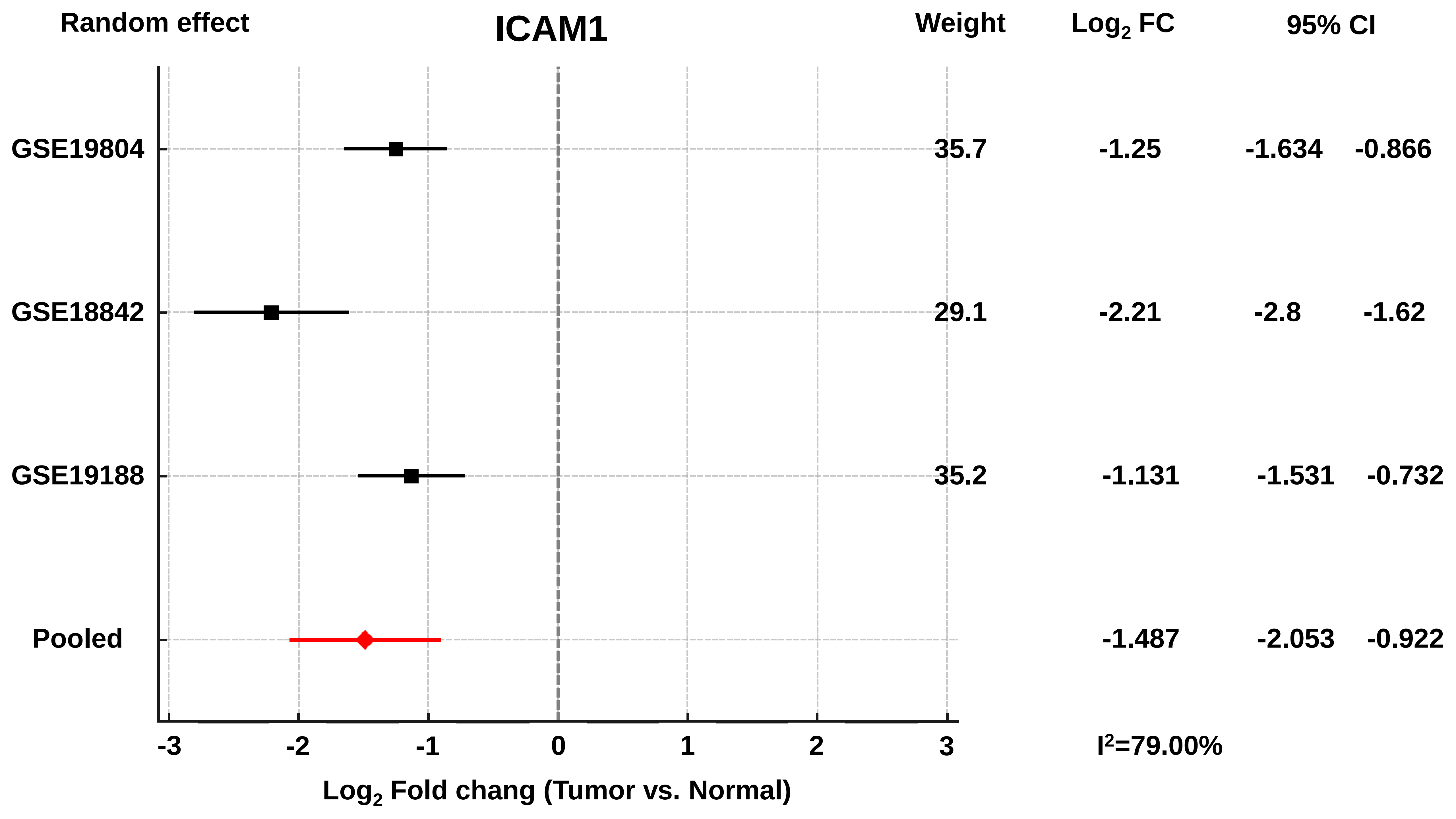
Random effect
ICAM1
Weight
Log2 FC
95% CI
GSE19804
35.7
-1.25
-1.634
-0.866
GSE18842
29.1
-2.21
-2.8
-1.62
GSE19188
35.2
-1.131
-1.531
-0.732
Pooled
-1.487
-2.053
-0.922
-3
-2
-1
0
1
2
3
I2=79.00%
Log2 Fold chang (Tumor vs. Normal)

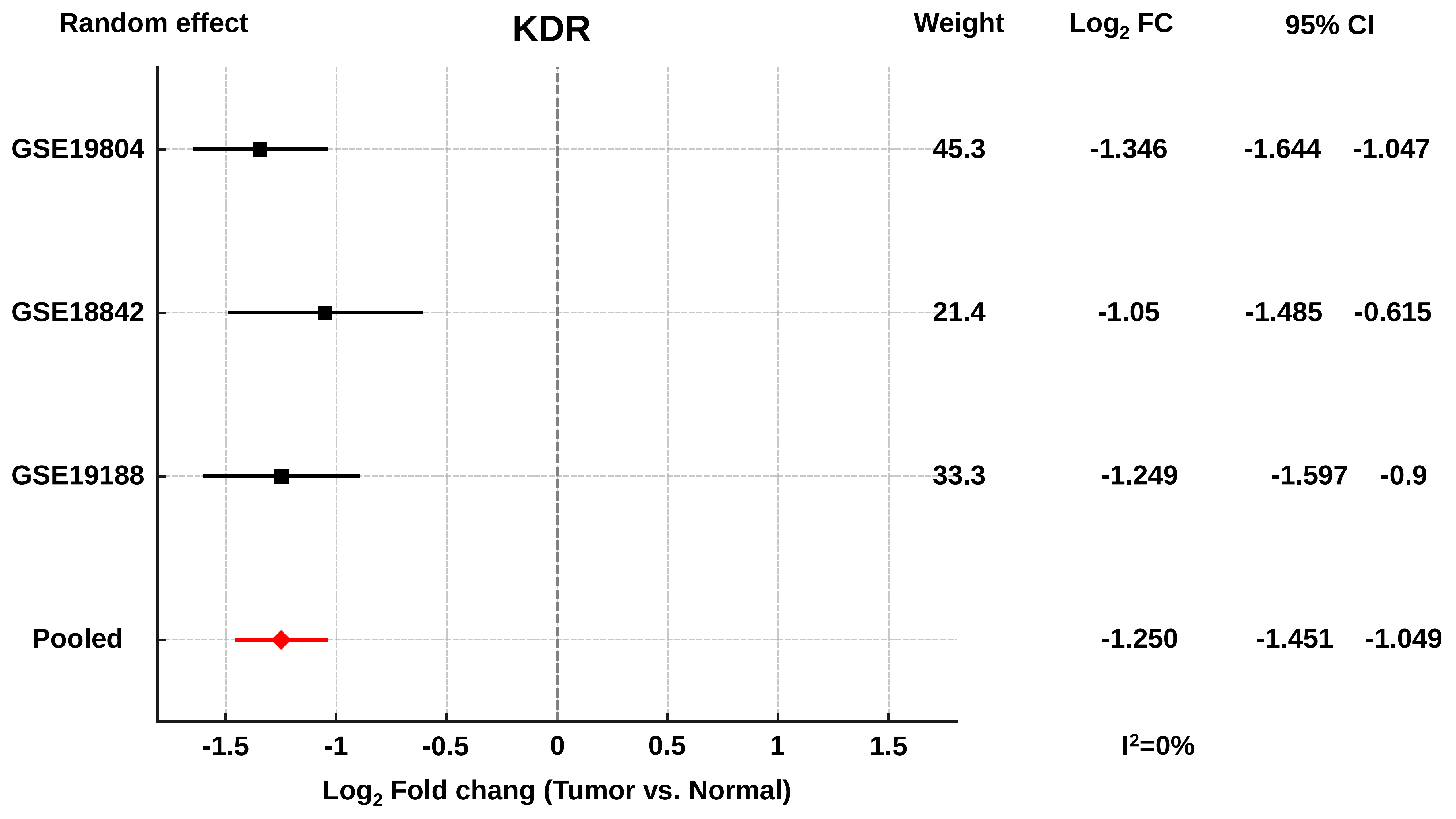
Random effect
KDR
Weight
Log2 FC
95% CI
GSE19804
45.3
-1.346
-1.644 -1.047
GSE18842
21.4
-1.05
-1.485 -0.615
GSE19188
33.3
-1.249
-1.597 -0.9
Pooled
-1.250
-1.451 -1.049
-1.5
-1
-0.5
0
0.5
1
1.5
I2=0%
Log2 Fold chang (Tumor vs. Normal)

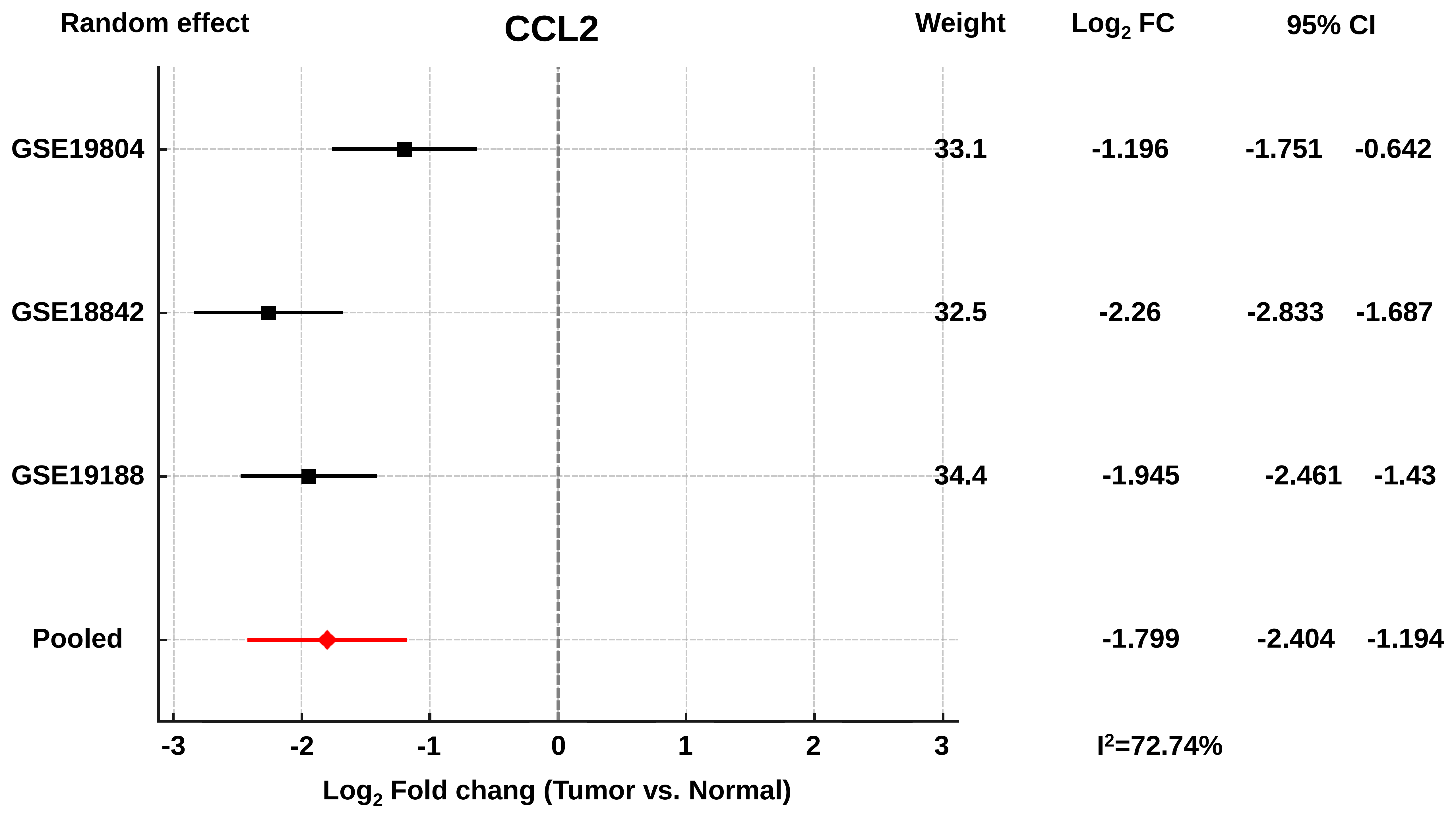

Random effect
CCL2
Weight
Log2 FC
95% CI
GSE19804
33.1
-1.196
-1.751
-0.642
GSE18842
32.5
-2.26
-2.833
-1.687
GSE19188
34.4
-1.945
-2.461
-1.43
Pooled
-1.799
-2.404
-1.194
-3
-2
-1
0
1
2
3
I2=72.74%
Log2 Fold chang (Tumor vs. Normal)

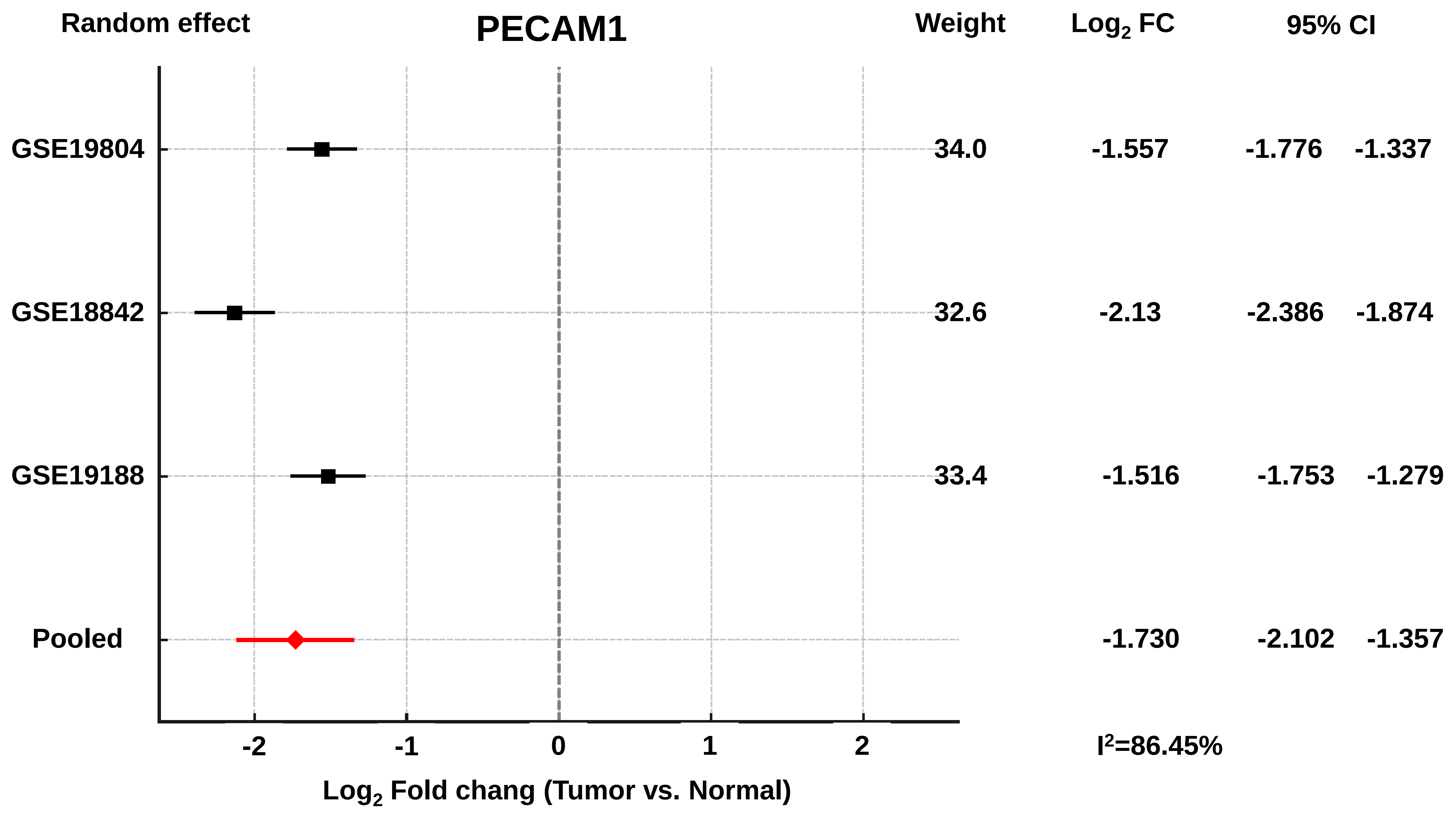
Random effect
PECAM1
Weight
Log2 FC
95% CI
GSE19804
34.0
-1.557
-1.776
-1.337
GSE18842
32.6
-2.13
-2.386
-1.874
GSE19188
33.4
-1.516
-1.753
-1.279
Pooled
-1.730
-2.102
-1.357
-2
-1
0
1
2
I2=86.45%
Log2 Fold chang (Tumor vs. Normal)

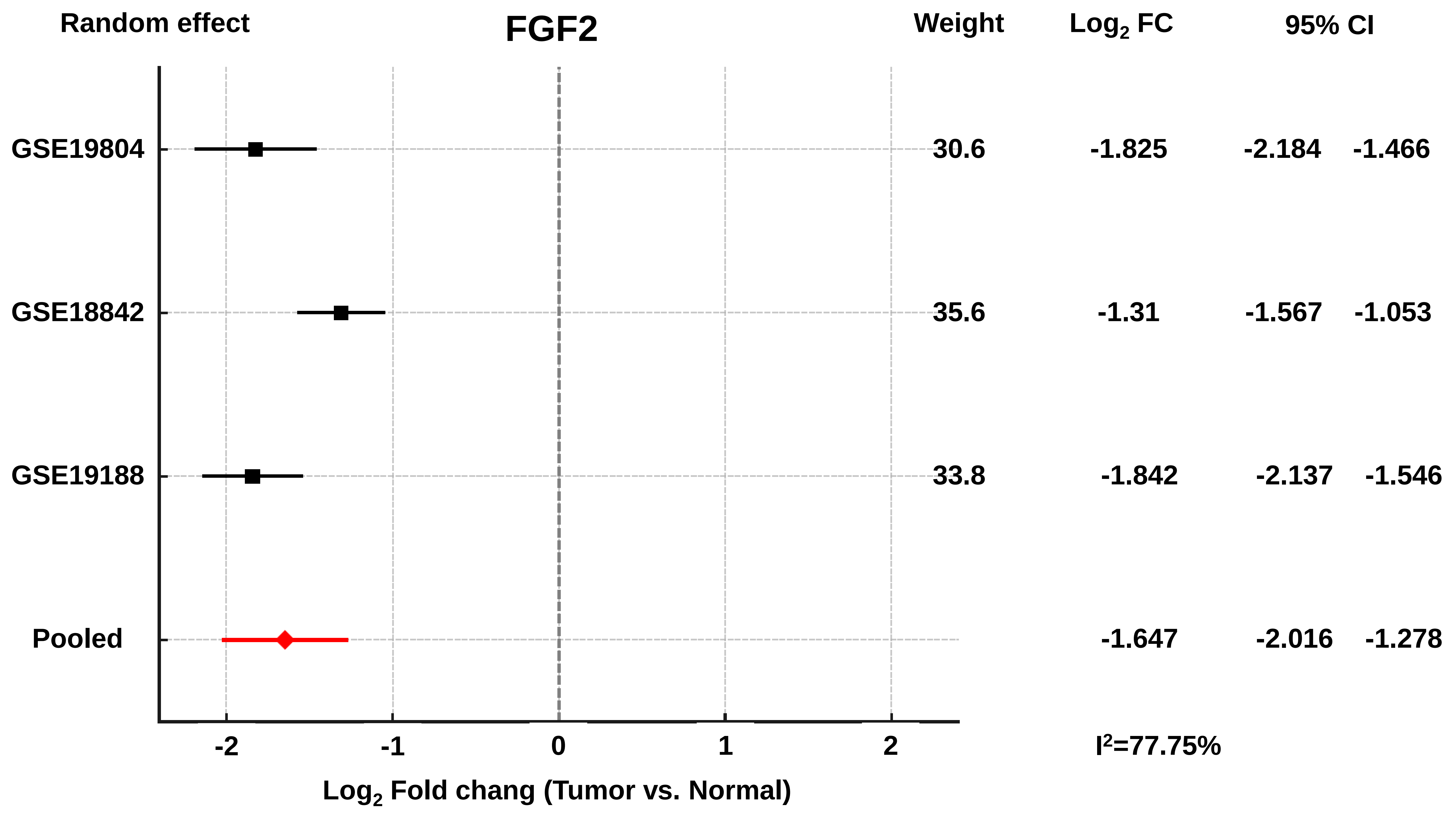
Random effect
FGF2
Weight
Log2 FC
95% CI
GSE19804
30.6
-1.825
-2.184
-1.466
GSE18842
35.6
-1.31
-1.567
-1.053
GSE19188
33.8
-1.842
-2.137
-1.546
Pooled
-1.647
-2.016
-1.278
-2
-1
0
1
2
I2=77.75%
Log2 Fold chang (Tumor vs. Normal)

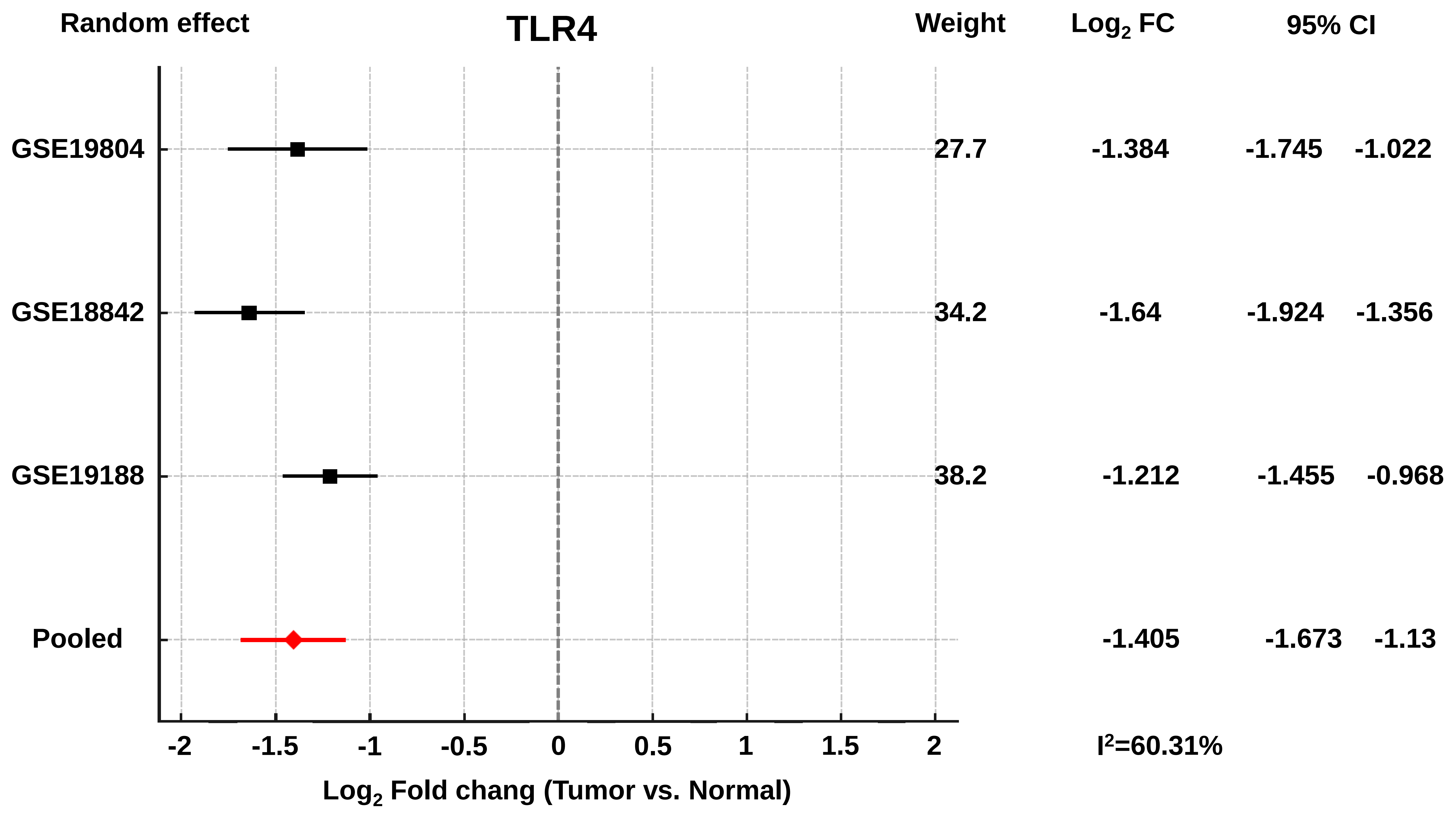

Random effect
TLR4
Weight
Log2 FC
95% CI
GSE19804
27.7
-1.384
-1.745
-1.022
GSE18842
34.2
-1.64
-1.924
-1.356
GSE19188
38.2
-1.212
-1.455
-0.968
Pooled
-1.405
-1.673
-1.13
-2
-1.5
-1
-0.5
0
0.5
1
1.5
2
I2=60.31%
Log2 Fold chang (Tumor vs. Normal)

Supplementary File S5.

Random-effects meta-analysis of differential expression effect sizes for the 11 hub genes. For each gene, the forest plot displays dataset-specific log_2 fold changes (log_2FC), standard errors, 95% confidence intervals, study weights, heterogeneity statistics (I^2), and pooled log_2FC estimates (random-effects model).
