# Supplementary material for: FGF2 as a Potential Tumor Suppressor in Lung Adenocarcinoma
Source: Diagnostics (Basel). 2026 Jan 13;16(2):250. doi: 10.3390/diagnostics16020250 (PMC12839716; doi:10.3390/diagnostics16020250)
Supplement: Supplementary file 1 [file diagnostics-16-00250-s001.zip › Supplementary Files S7.pdf]

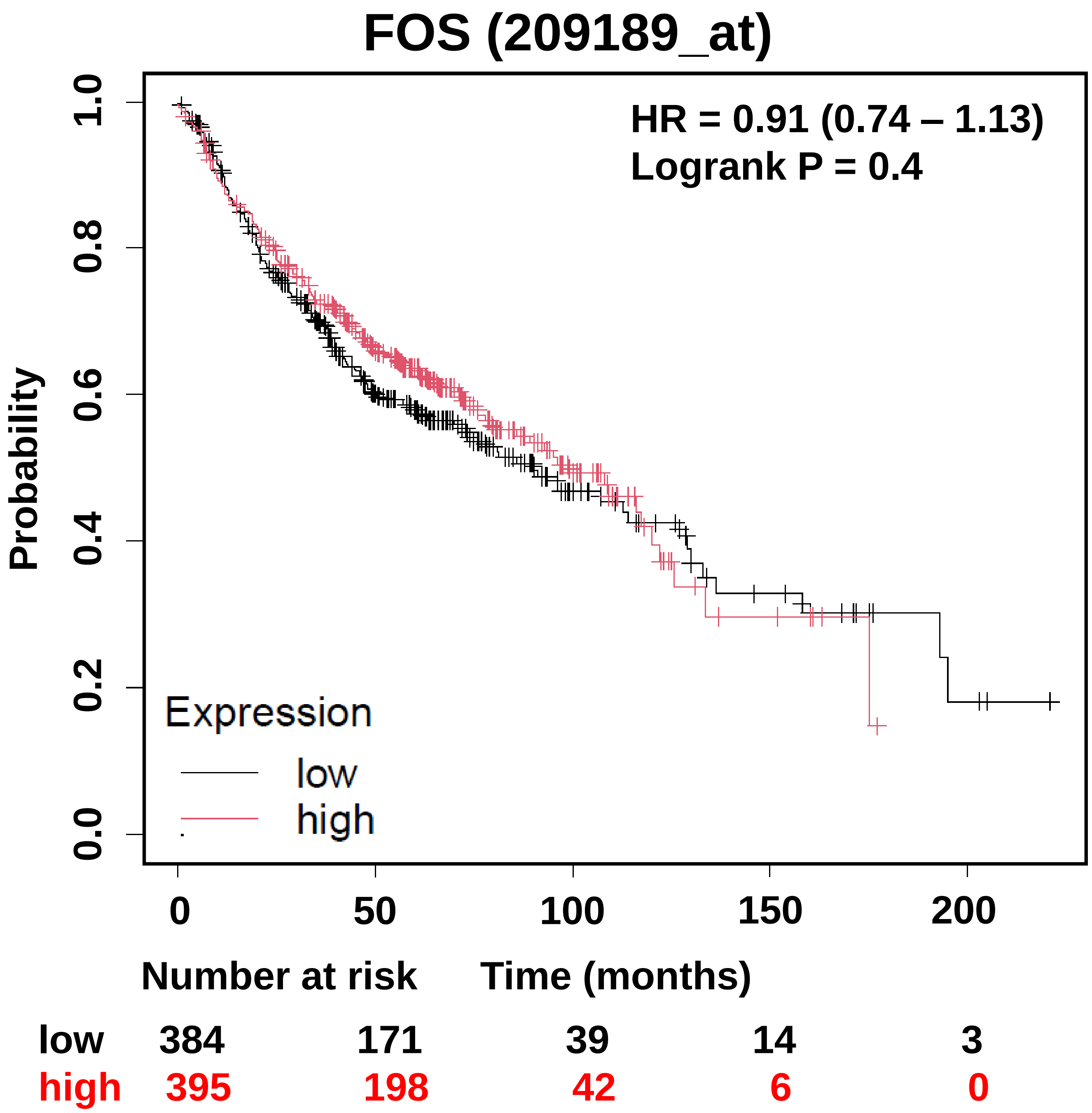
FOS (209189_at)
HR = 0.91 (0.74 – 1.13)
Logrank P = 0.4
Probability
1.0
0.8
0.6
0.4
0.2
0.0
Expression
low
high
0
50
100
150
200
Number at risk
Time (months)
low 384 171 39 14 3
high 395 198 42 6 0

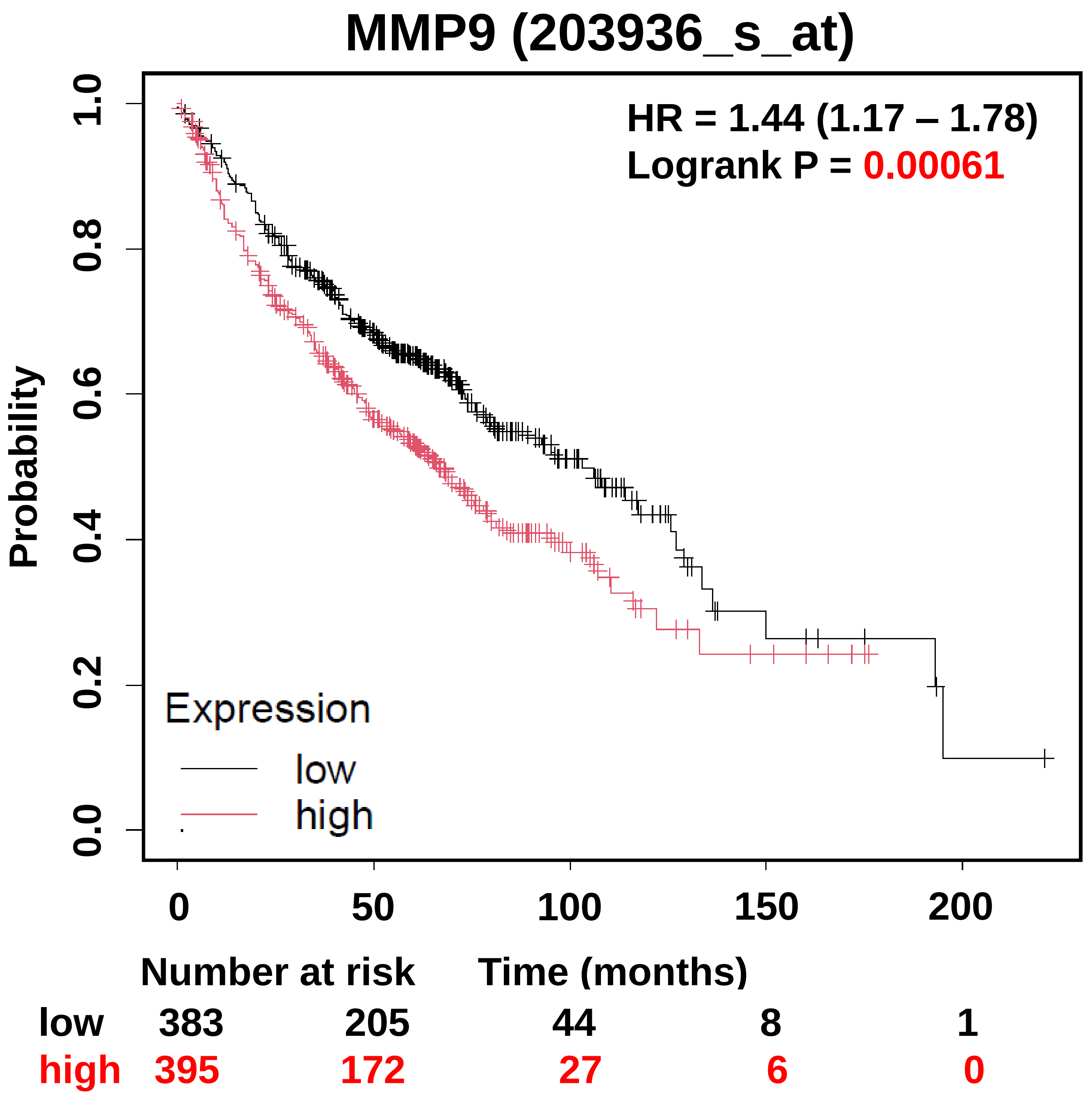
MMP9 (203936_s_at)
HR = 1.44 (1.17 – 1.78)
Logrank P = 0.00061
Probability
0.0
0.2
0.4
0.6
0.8
1.0
Expression
low
high
0
50
100
150
200
Number at risk
Time (months)
low 383 205 44 8 1
high 395 172 27 6 0

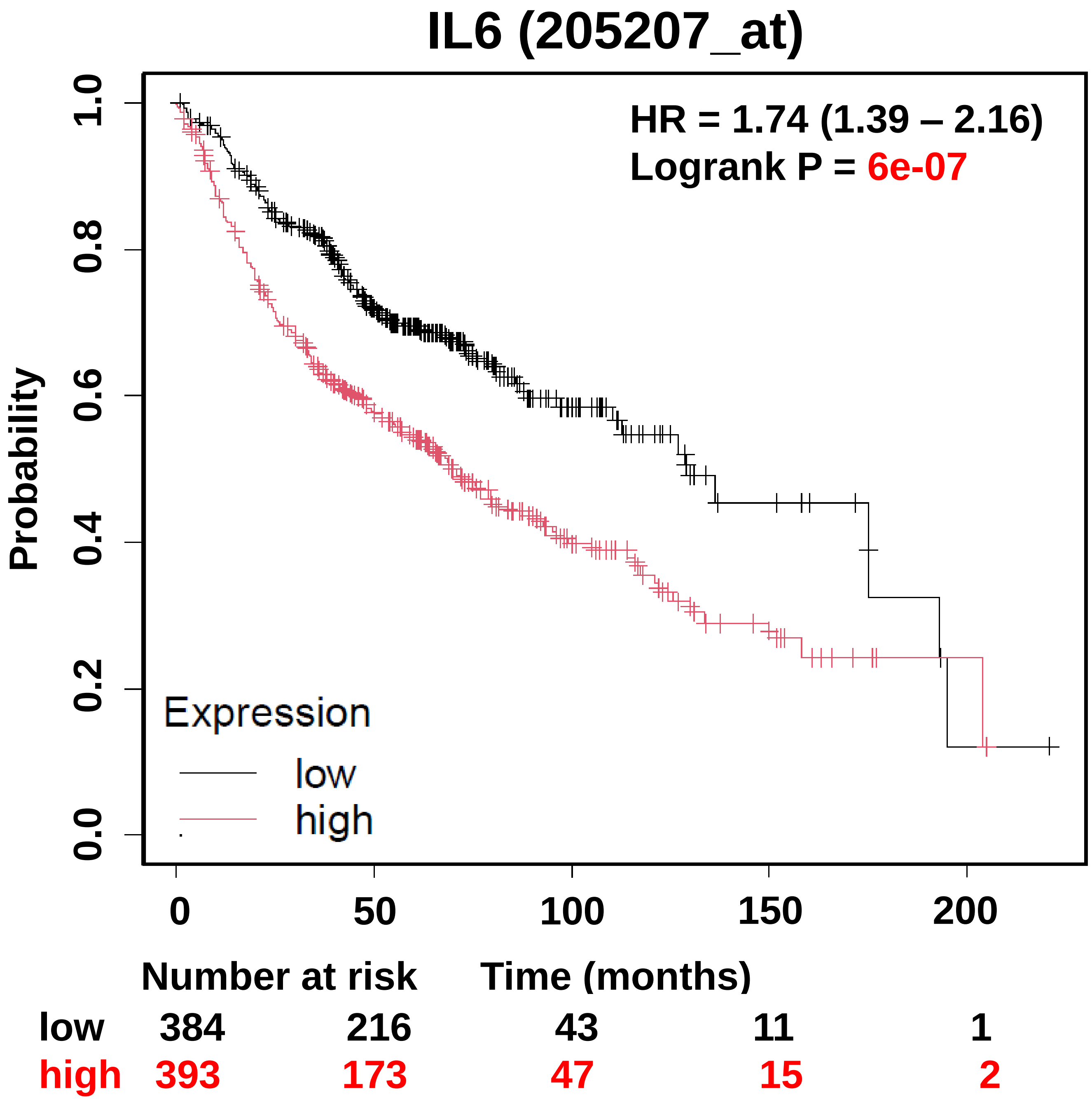
IL6 (205207_at)
HR = 1.74 (1.39 – 2.16)
Logrank P = 6e-07
Probability
0.0
0.2
0.4
0.6
0.8
1.0
Expression
low
high
0
50
100
150
200
Number at risk
Time (months)
low 384 216 43 11 1
high 393 173 47 15 2

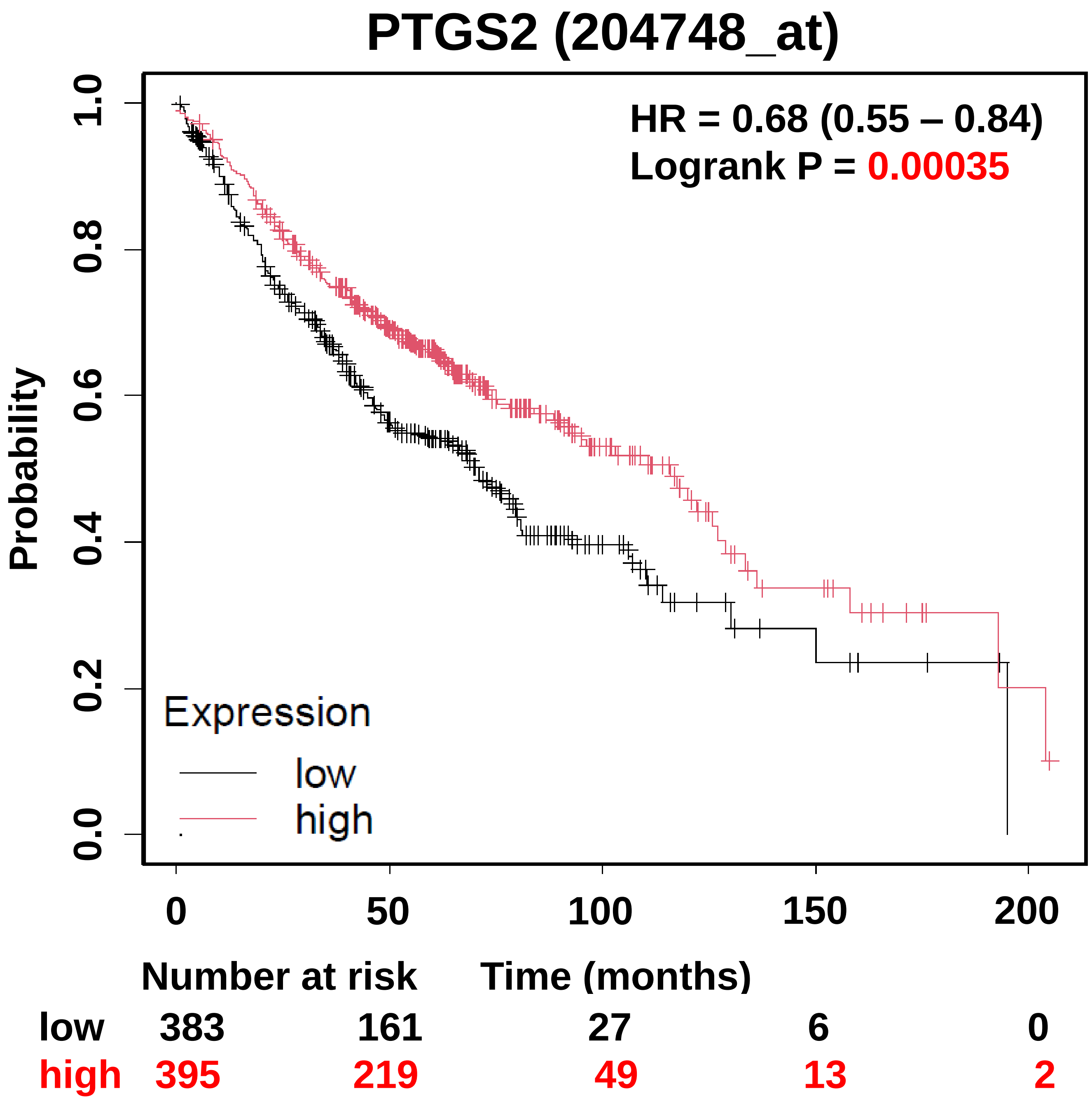
PTGS2 (204748_at)
HR = 0.68 (0.55 – 0.84)
Logrank P = 0.00035
Probability
1.0
0.8
0.6
0.4
0.2
0.0
Expression
low
high
0
50
100
150
200
Number at risk
Time (months)
low 383 161 27 6 0
high 395 219 49 13 2

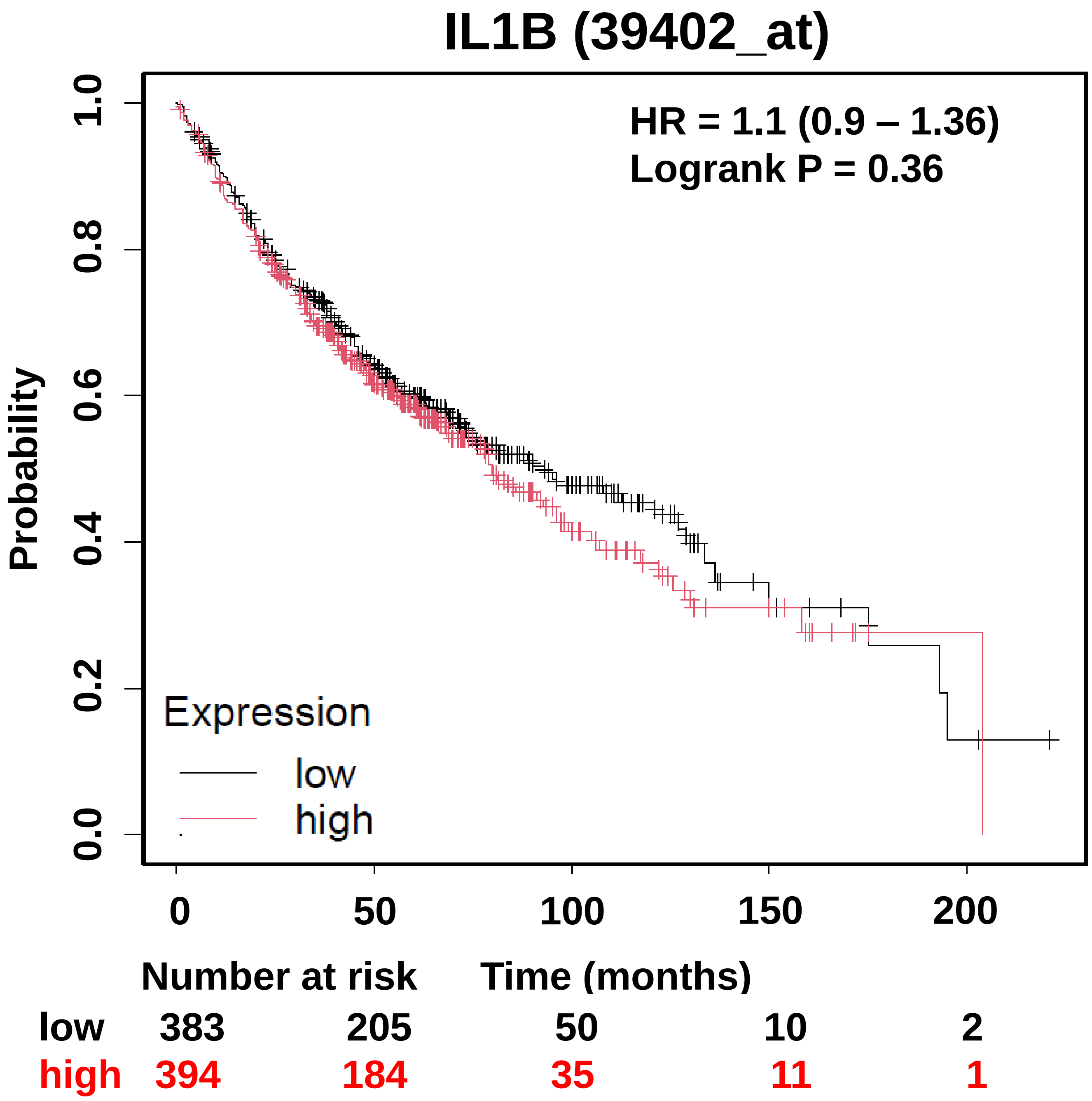
IL1B (39402_at)
HR = 1.1 (0.9 – 1.36)
Logrank P = 0.36
Probability
0.0
0.2
0.4
0.6
0.8
1.0
Expression
low
high
0
50
100
150
200
Number at risk
Time (months)
low 383 205 50 10 2
high 394 184 35 11 1

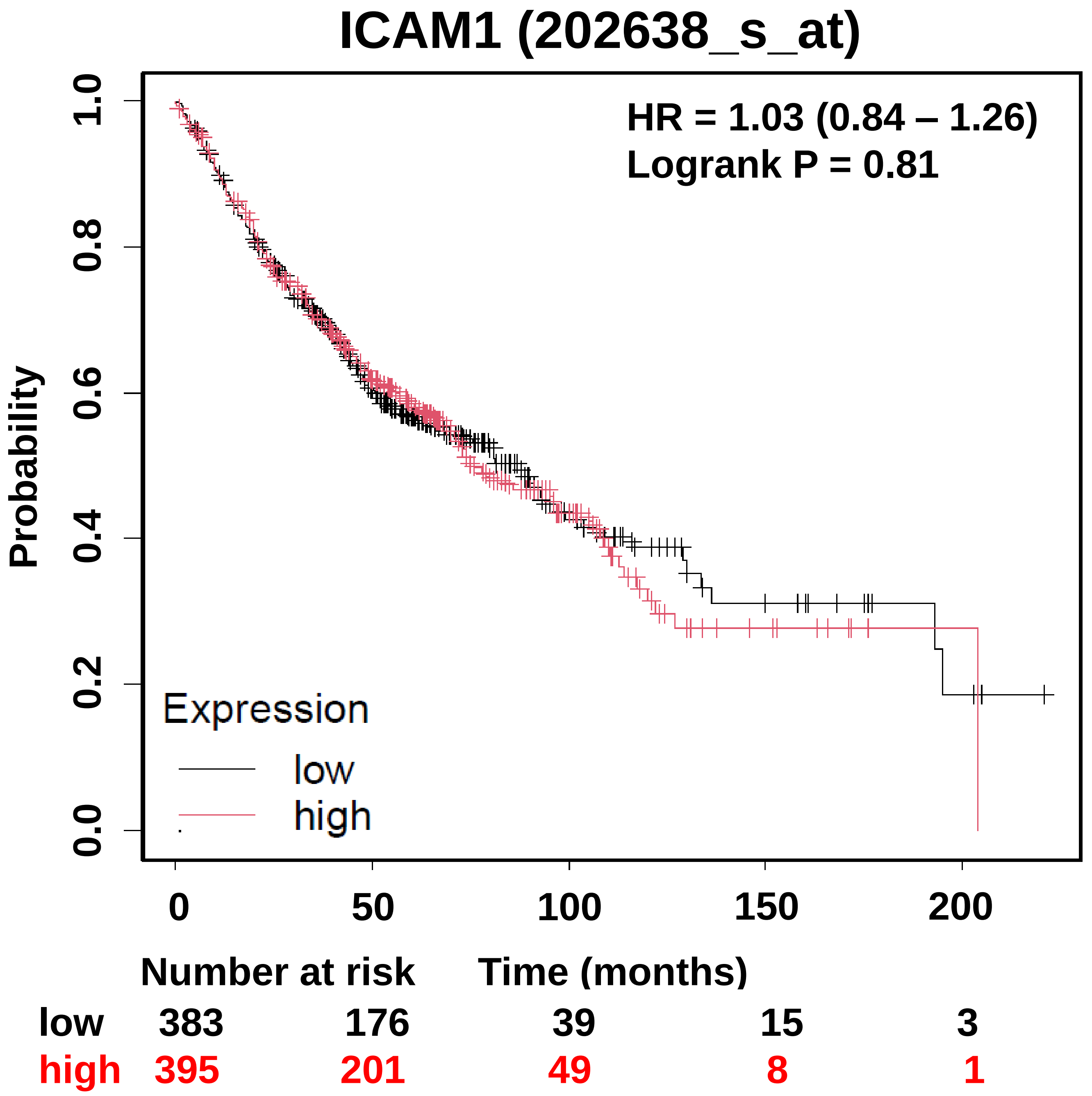
ICAM1 (202638_s_at)
HR = 1.03 (0.84 – 1.26)
Logrank P = 0.81
Probability
0.0
0.2
0.4
0.6
0.8
1.0
Expression
low
high
0
50
100
150
200
Number at risk
Time (months)
low 383 176 39 15 3
high 395 201 49 8 1

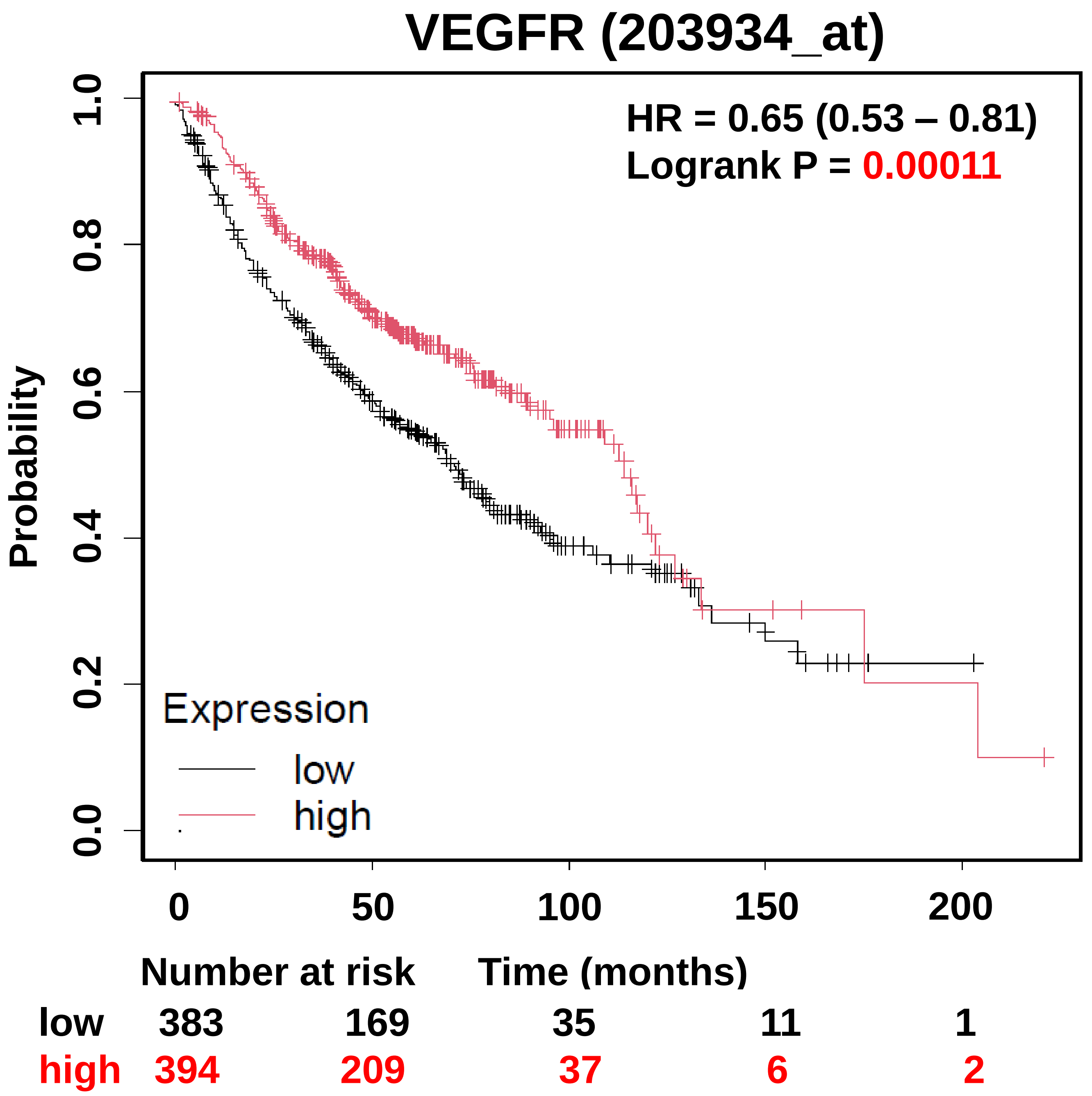
VEGFR (203934_at)
HR = 0.65 (0.53 – 0.81)
Logrank P = 0.00011
Probability
0.0
0.2
0.4
0.6
0.8
1.0
Expression
low
high
0
50
100
150
200
Number at risk
Time (months)
low 383 169 35 11 1
high 394 209 37 6 2

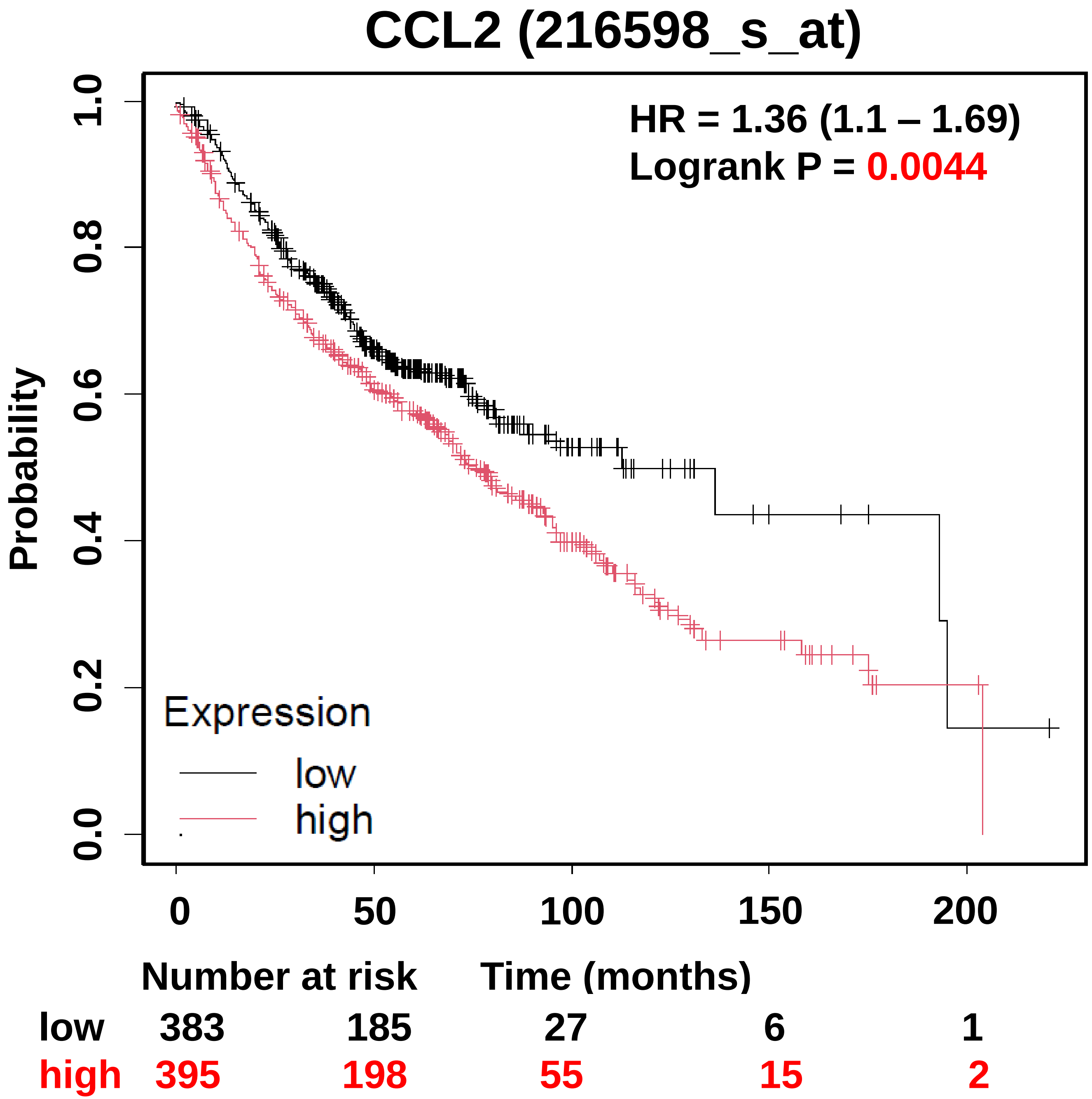
CCL2 (216598_s_at)
HR = 1.36 (1.1 – 1.69)
Logrank P = 0.0044
Probability
0.0
0.2
0.4
0.6
0.8
1.0
Expression
low
high
0
50
100
150
200
Number at risk
Time (months)
low 383 185 27 6 1
high 395 198 55 15 2

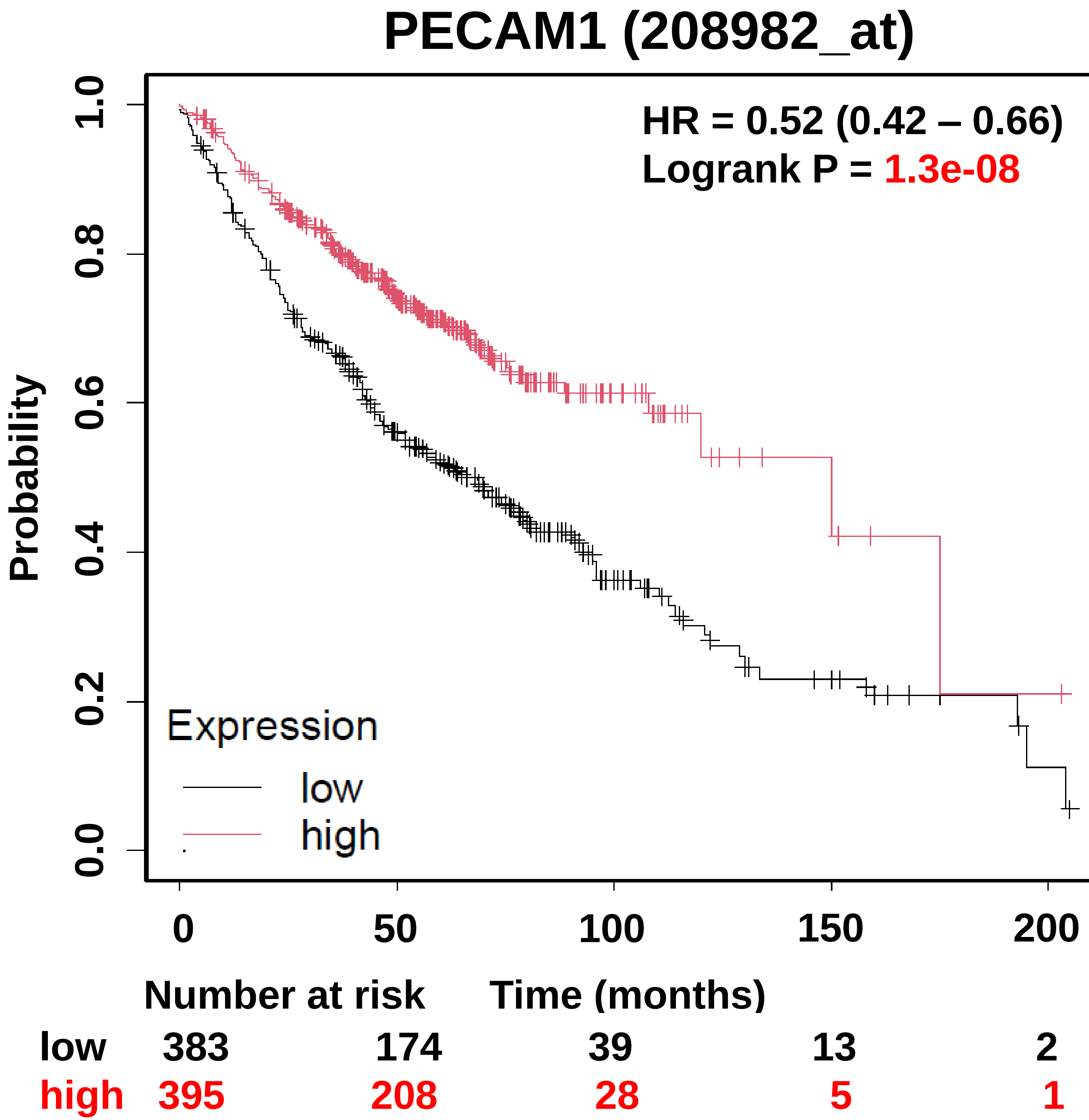
PECAM1 (208982_at)
HR = 0.52 (0.42 – 0.66)
Logrank P = 1.3e-08
Probability
1.0
0.8
0.6
0.4
0.2
0.0
Expression
low
high
0
50
100
150
200
Number at risk
Time (months)
low 383 174 39 13 2
high 395 208 28 5 1

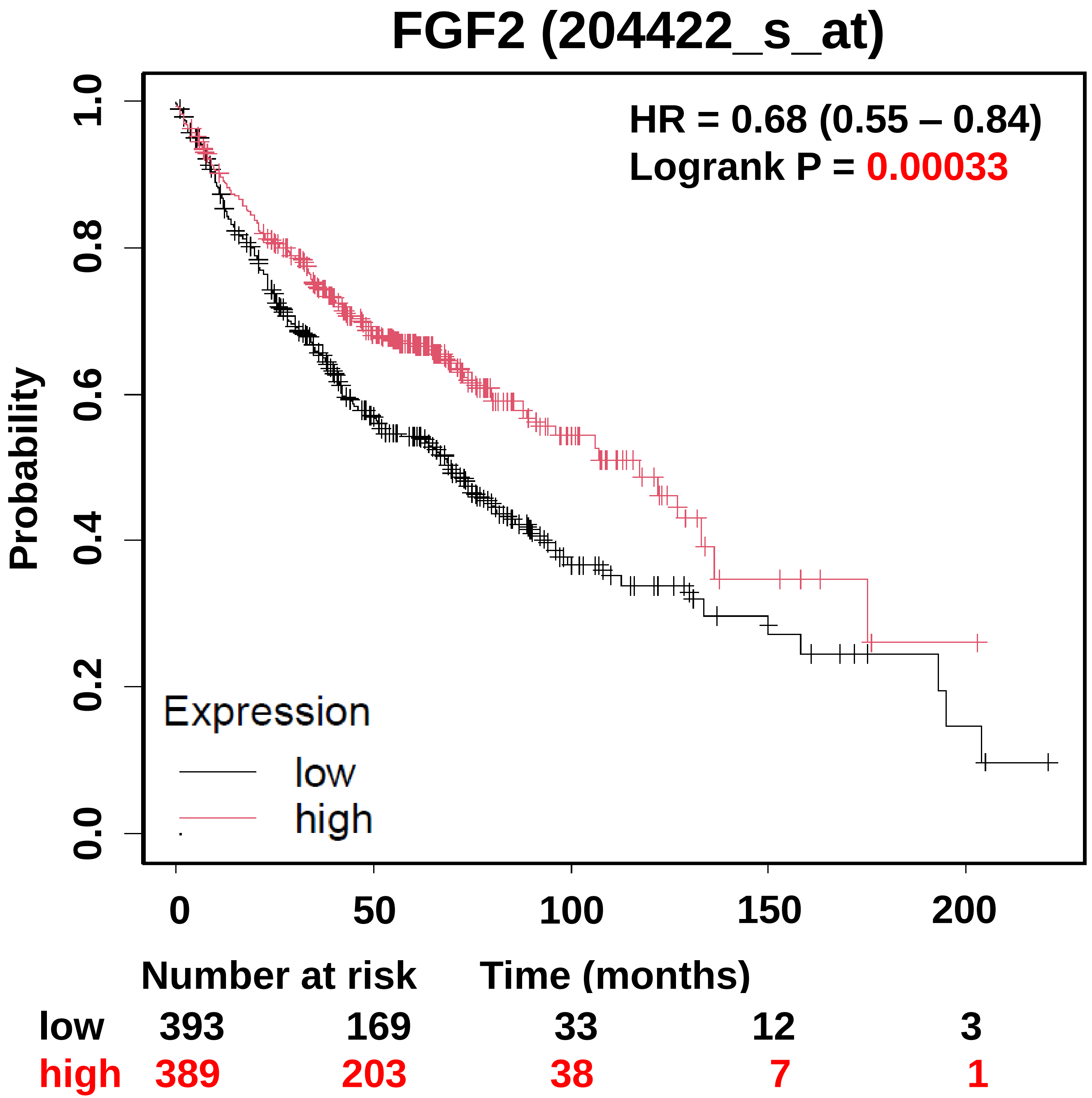

FGF2 (204422_s_at)
HR = 0.68 (0.55 – 0.84)
Logrank P = 0.00033
Probability
0.0
0.2
0.4
0.6
0.8
1.0
Expression
low
high
0
50
100
150
200
Number at risk
Time (months)
low 393 169 33 12 3
high 389 203 38 7 1

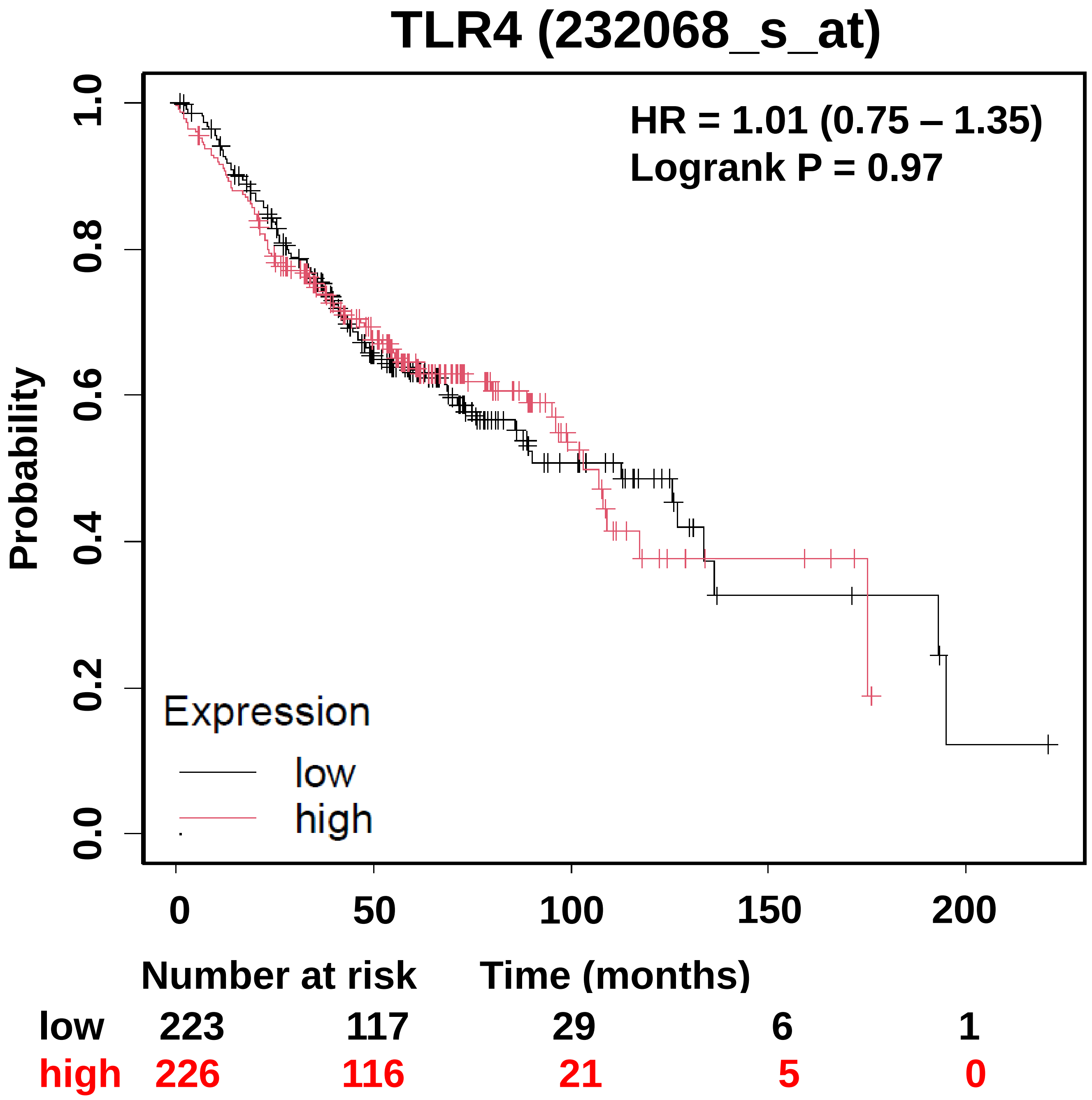
TLR4 (232068_s_at)
HR = 1.01 (0.75 – 1.35)
Logrank P = 0.97
Probability
0.0
0.2
0.4
0.6
0.8
1.0
Expression
low
high
0
50
100
150
200
Number at risk
Time (months)
low 223 117 29 6 1
high 226 116 21 5 0

**Overall survival (OS) analysis of hub genes in LUAD patients.** To avoid significance inflation associated with the KM-Plotter “auto select best cutoff,” survival analyses were also proceeded to use predefined expression tertiles. For each hub gene, patients were split into T1 (low expression) and T3 (high expression) groups, and univariate Cox proportional hazards models were used to estimate HRs and 95% confi-dence intervals for overall survival (OS) and first progression (FP).

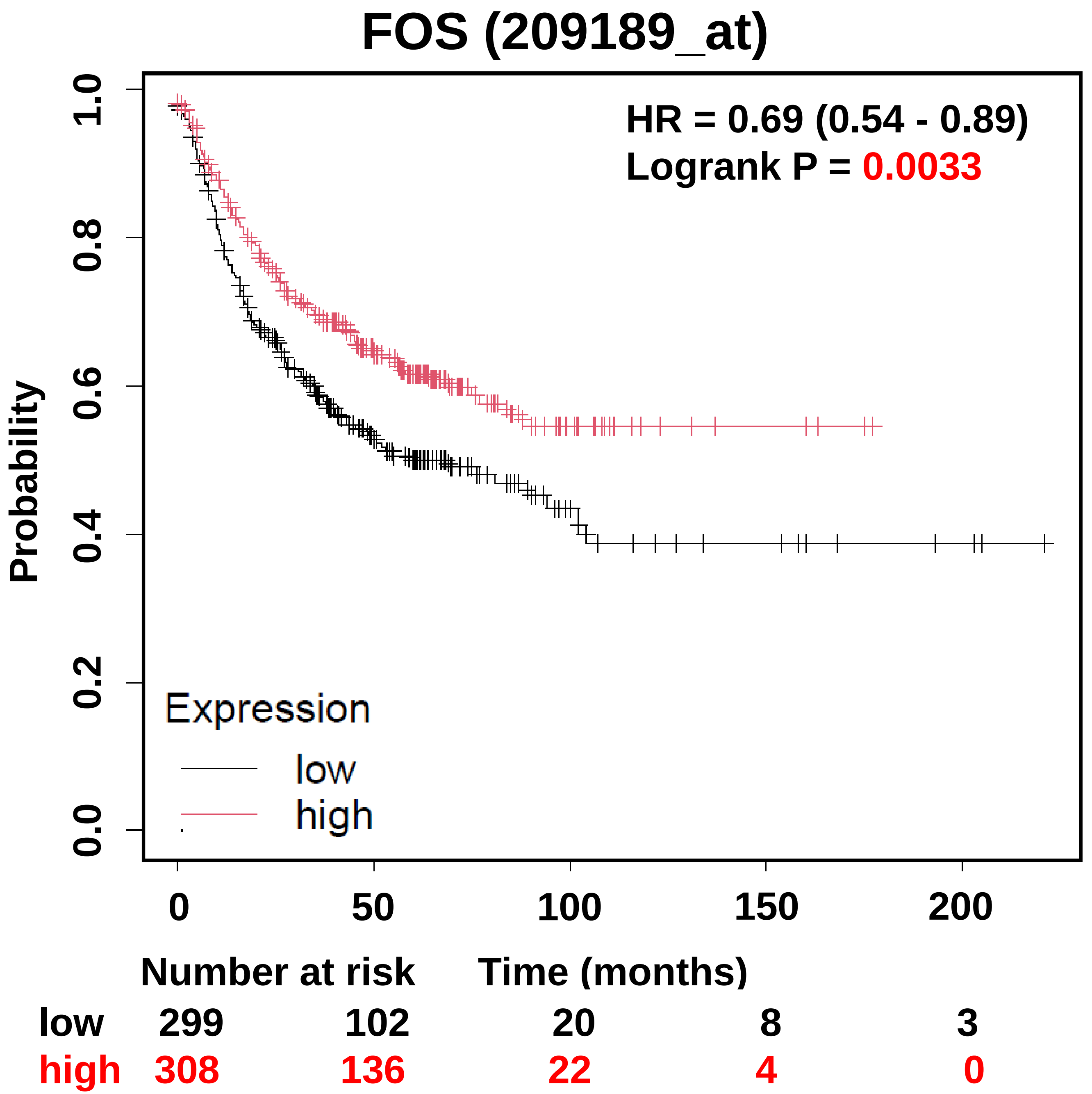

FOS (209189_at)
HR = 0.69 (0.54 - 0.89)
Logrank P = 0.0033
Probability
0.0
0.2
0.4
0.6
0.8
1.0
Expression
low
high
0
50
100
150
200
Number at risk
Time (months)
low 299 102 20 8 3
high 308 136 22 4 0

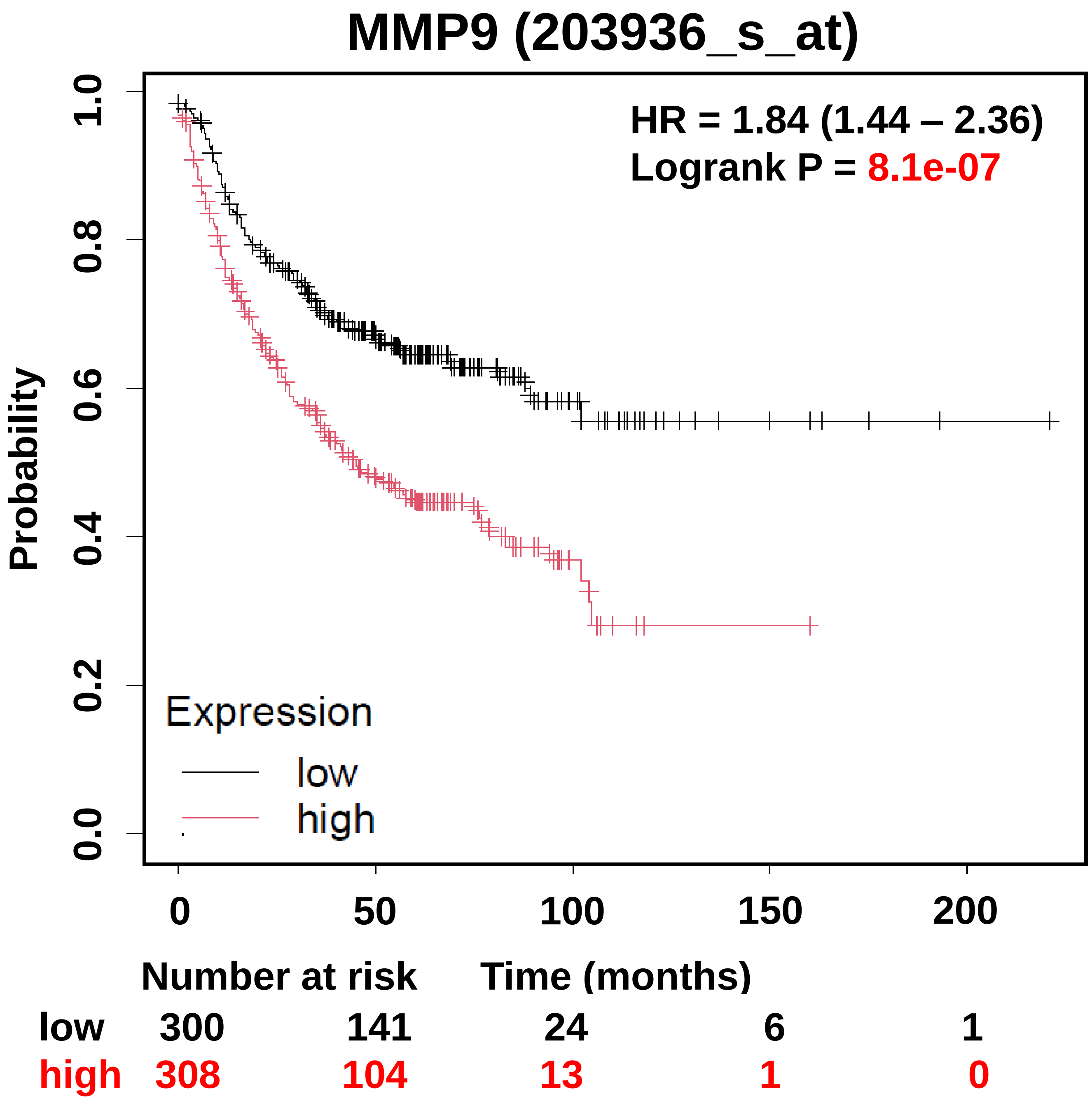
MMP9 (203936_s_at)
HR = 1.84 (1.44 – 2.36)
Logrank P = 8.1e-07
Probability
0.0
0.2
0.4
0.6
0.8
1.0
Expression
low
high
0
50
100
150
200
Number at risk
Time (months)
low 300 141 24 6 1
high 308 104 13 1 0

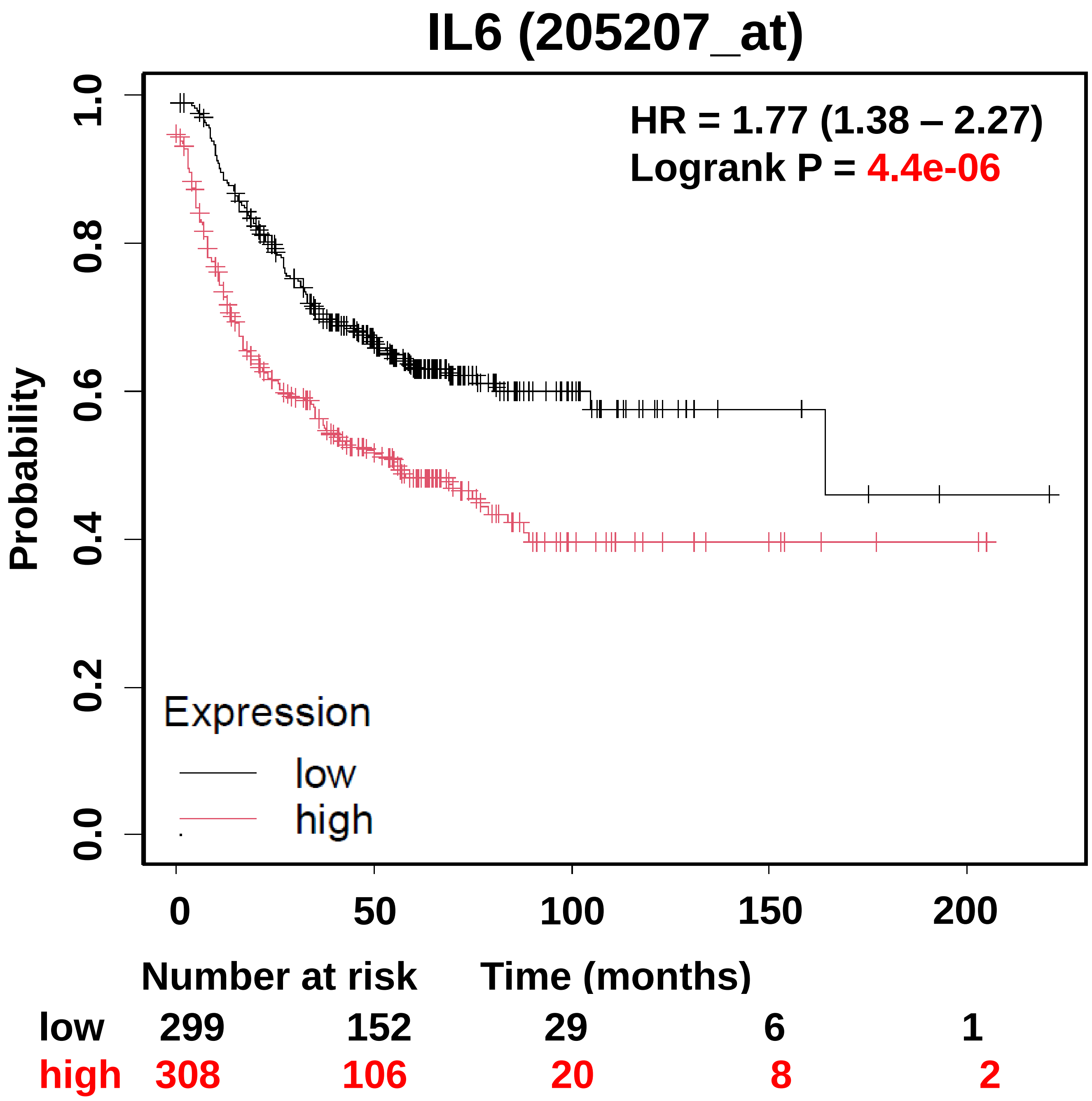
IL6 (205207_at)
HR = 1.77 (1.38 – 2.27)
Logrank P = 4.4e-06
Probability
0.0
0.2
0.4
0.6
0.8
1.0
Expression
low
high
0
50
100
150
200
Number at risk
Time (months)
low 299 152 29 6 1
high 308 106 20 8 2

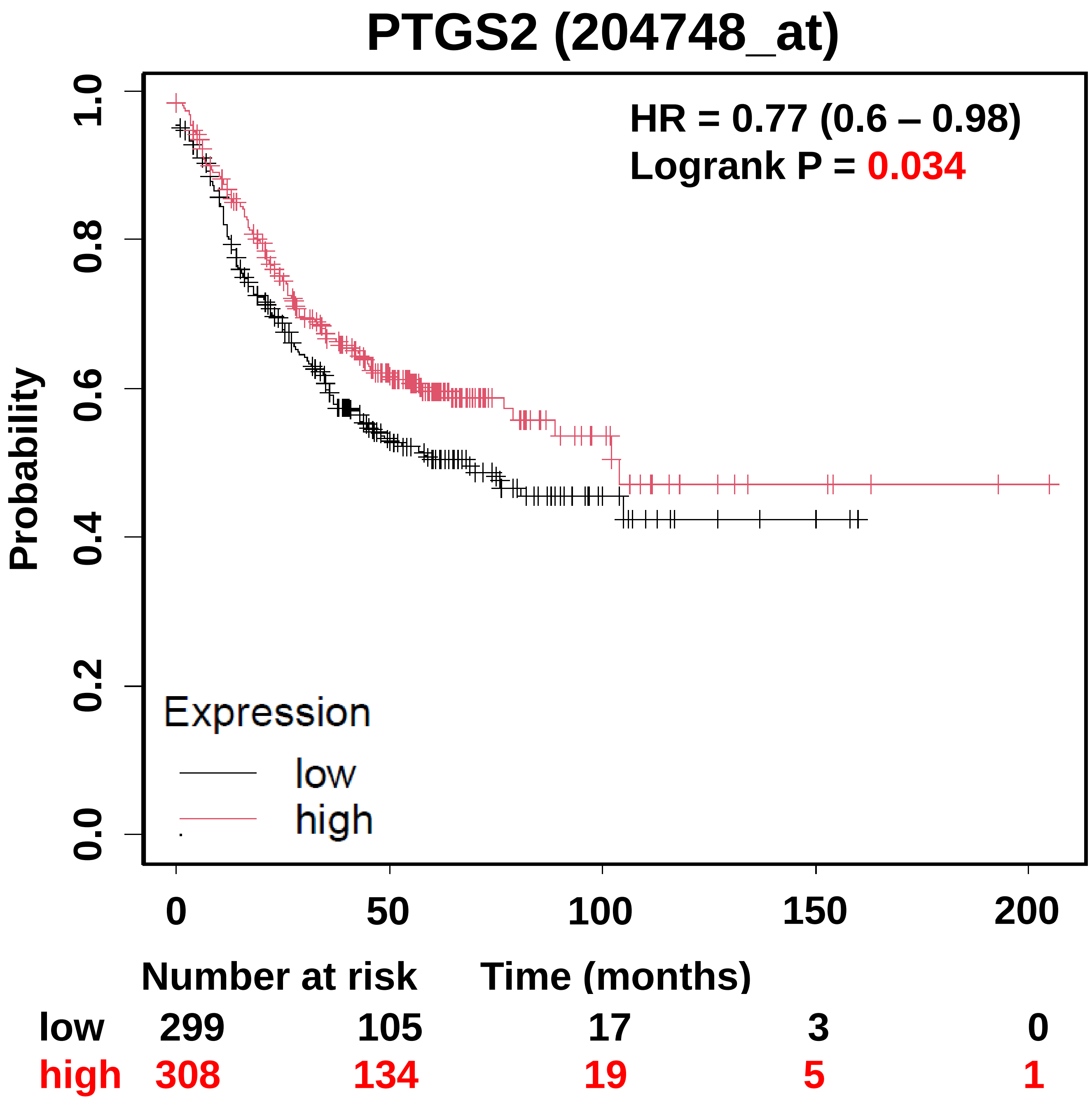
PTGS2 (204748_at)
HR = 0.77 (0.6 – 0.98)
Logrank P = 0.034
Probability
1.0
0.8
0.6
0.4
0.2
0.0
Expression
low
high
0
50
100
150
200
Number at risk
Time (months)
low 299 105 17 3 0
high 308 134 19 5 1

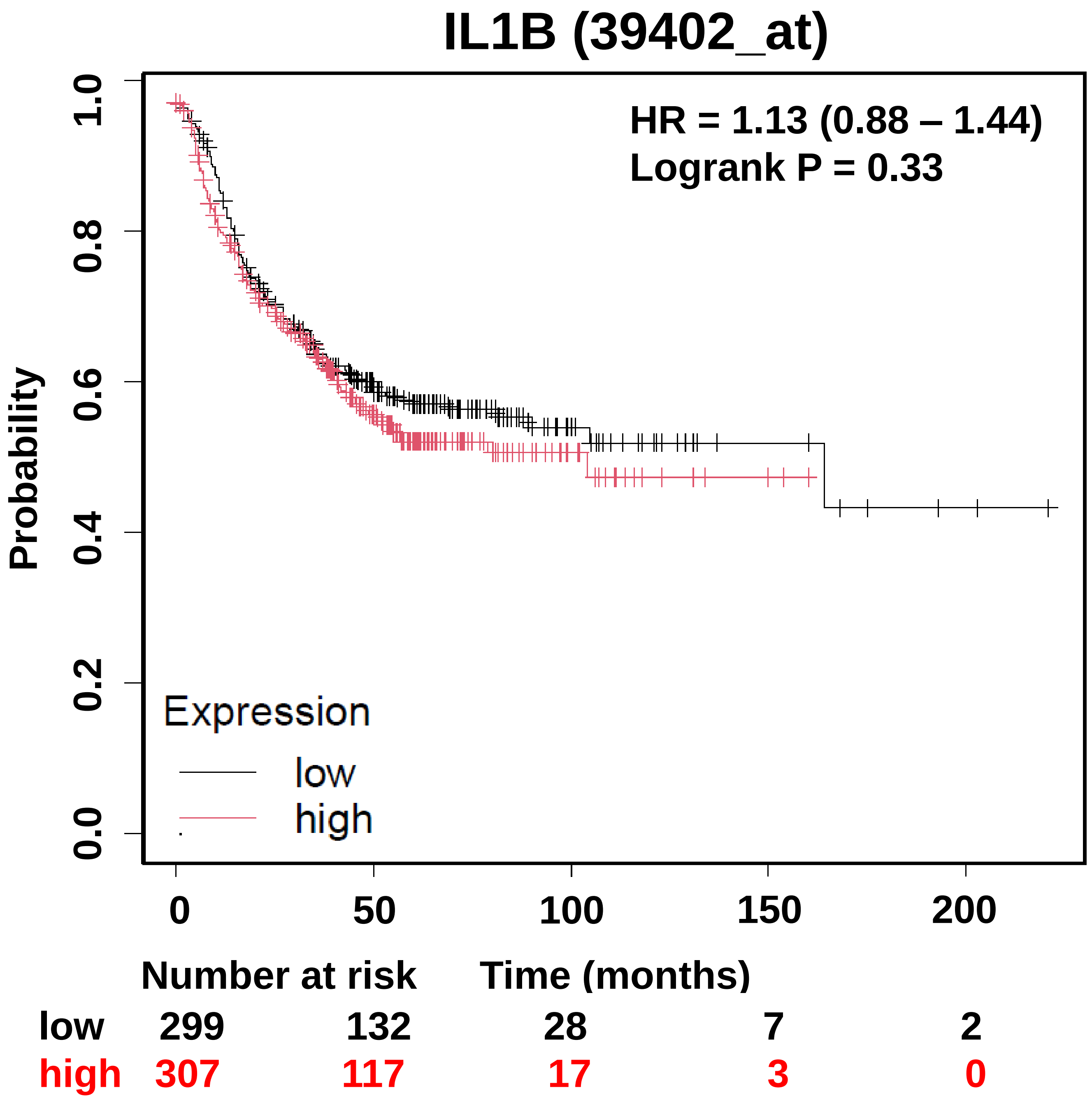
IL1B (39402_at)
HR = 1.13 (0.88 – 1.44)
Logrank P = 0.33
Probability
1.0
0.8
0.6
0.4
0.2
0.0
Expression
low
high
0
50
100
150
200
Number at risk
Time (months)
low 299 132 28 7 2
high 307 117 17 3 0

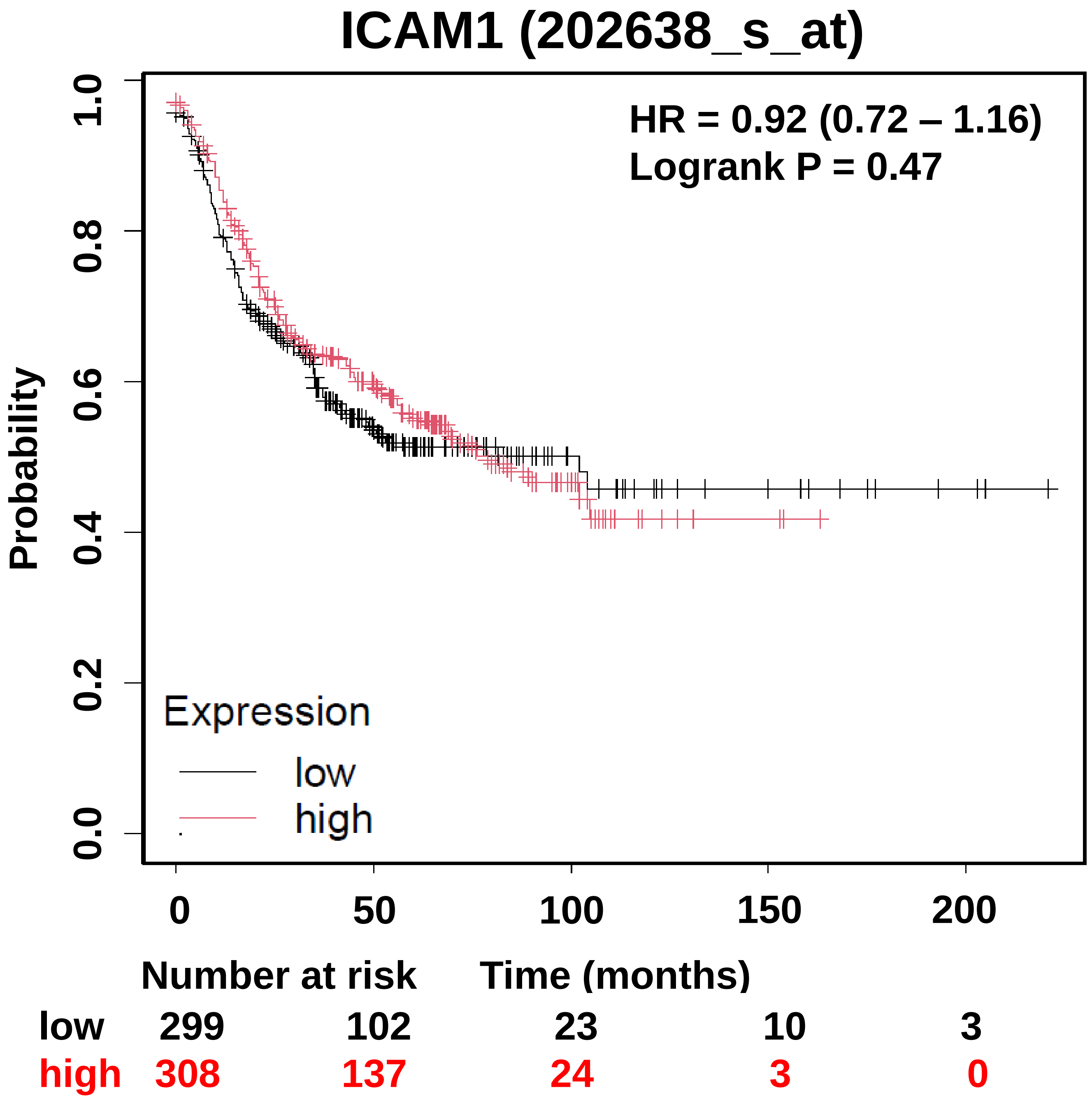

ICAM1 (202638_s_at)
HR = 0.92 (0.72 – 1.16)
Logrank P = 0.47
Probability
0.0
0.2
0.4
0.6
0.8
1.0
Expression
low
high
0
50
100
150
200
Number at risk
Time (months)
low 299 102 23 10 3
high 308 137 24 3 0

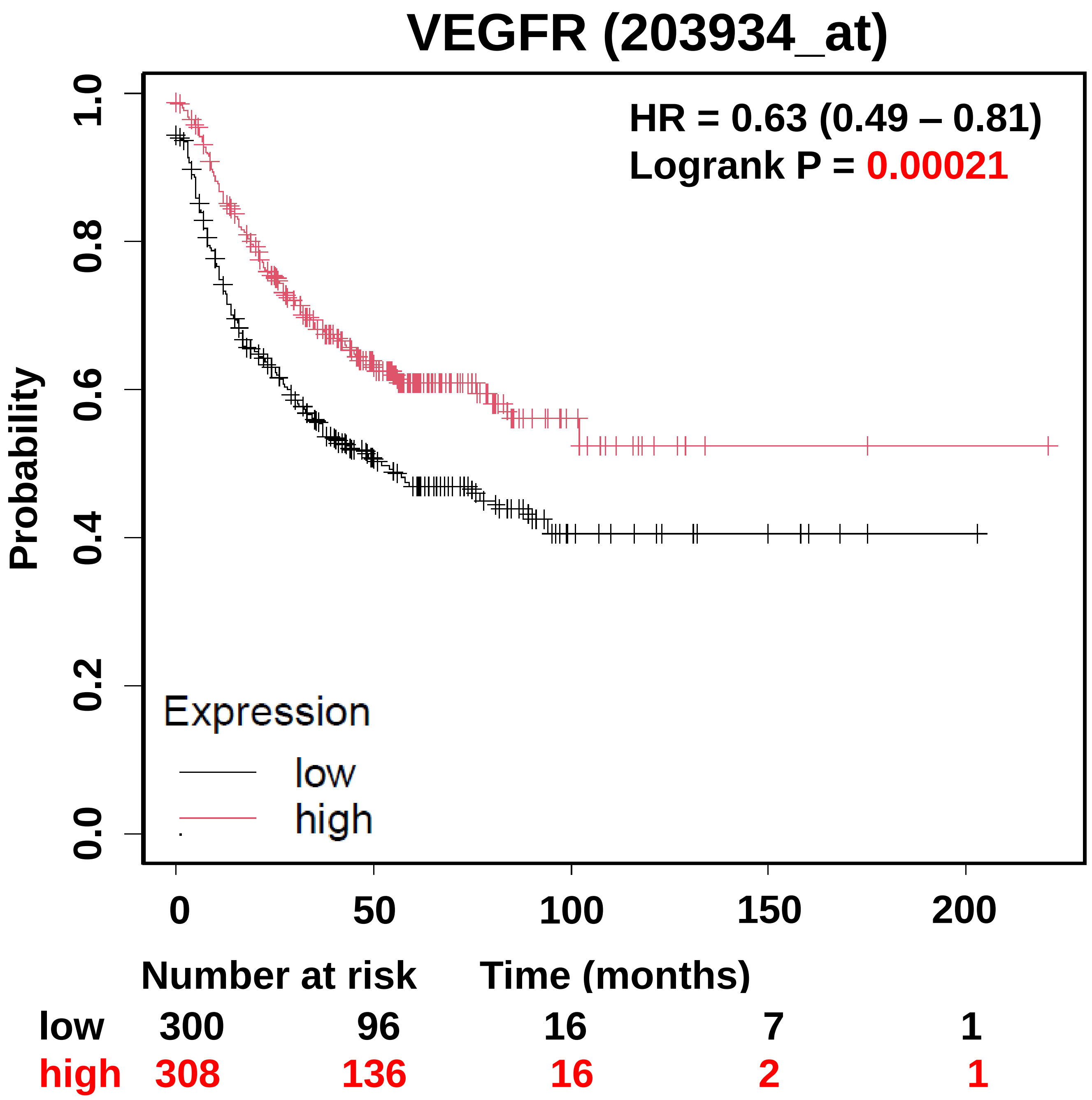

VEGFR (203934_at)
HR = 0.63 (0.49 – 0.81)
Logrank P = 0.00021
Probability
0.0
0.2
0.4
0.6
0.8
1.0
Expression
low
high
0
50
100
150
200
Number at risk
Time (months)
low 300 96 16 7 1
high 308 136 16 2 1

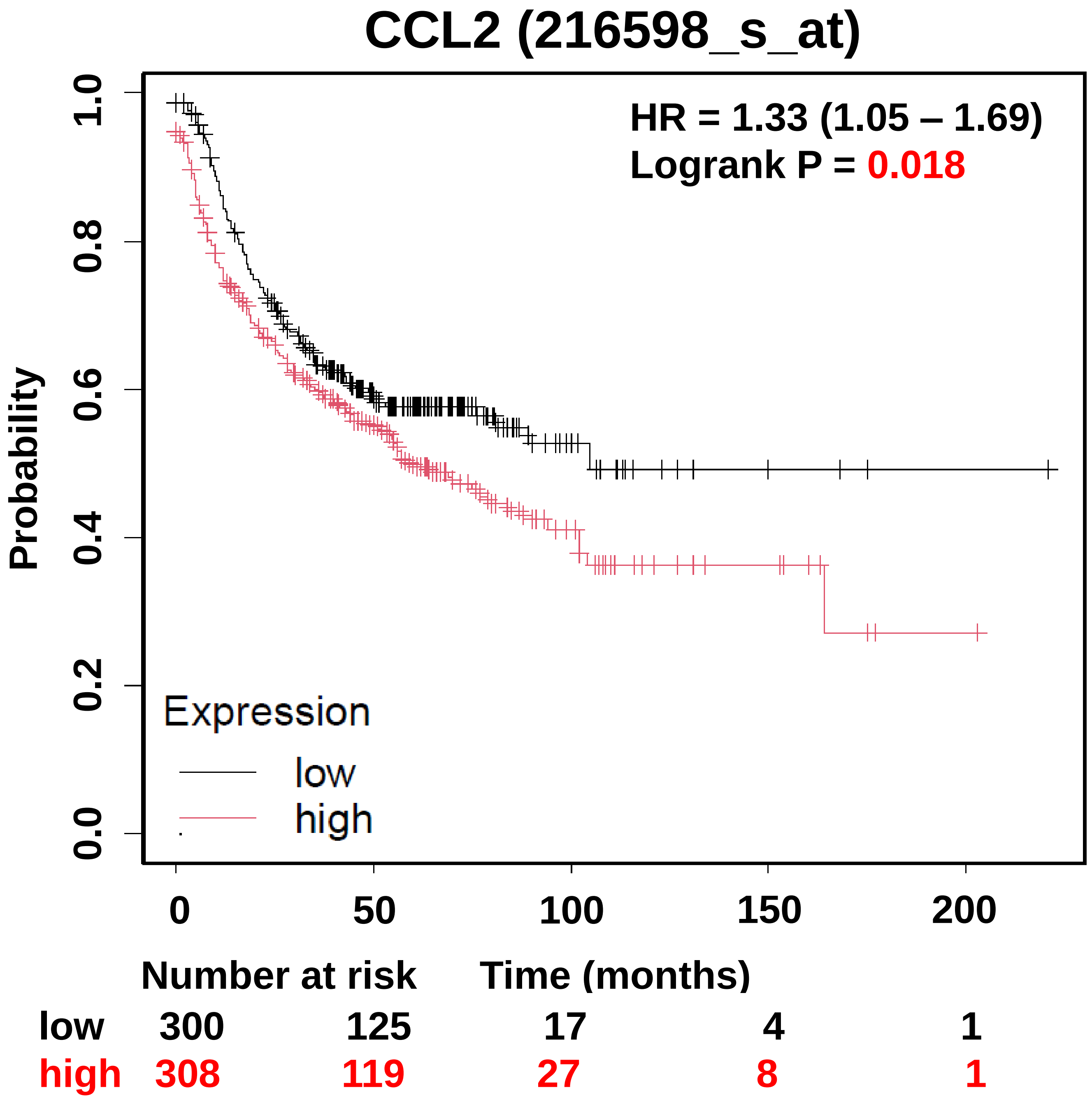
CCL2 (216598_s_at)
HR = 1.33 (1.05 – 1.69)
Logrank P = 0.018
Probability
0.0
0.2
0.4
0.6
0.8
1.0
Expression
low
high
0
50
100
150
200
Number at risk
Time (months)
low 300 125 17 4 1
high 308 119 27 8 1

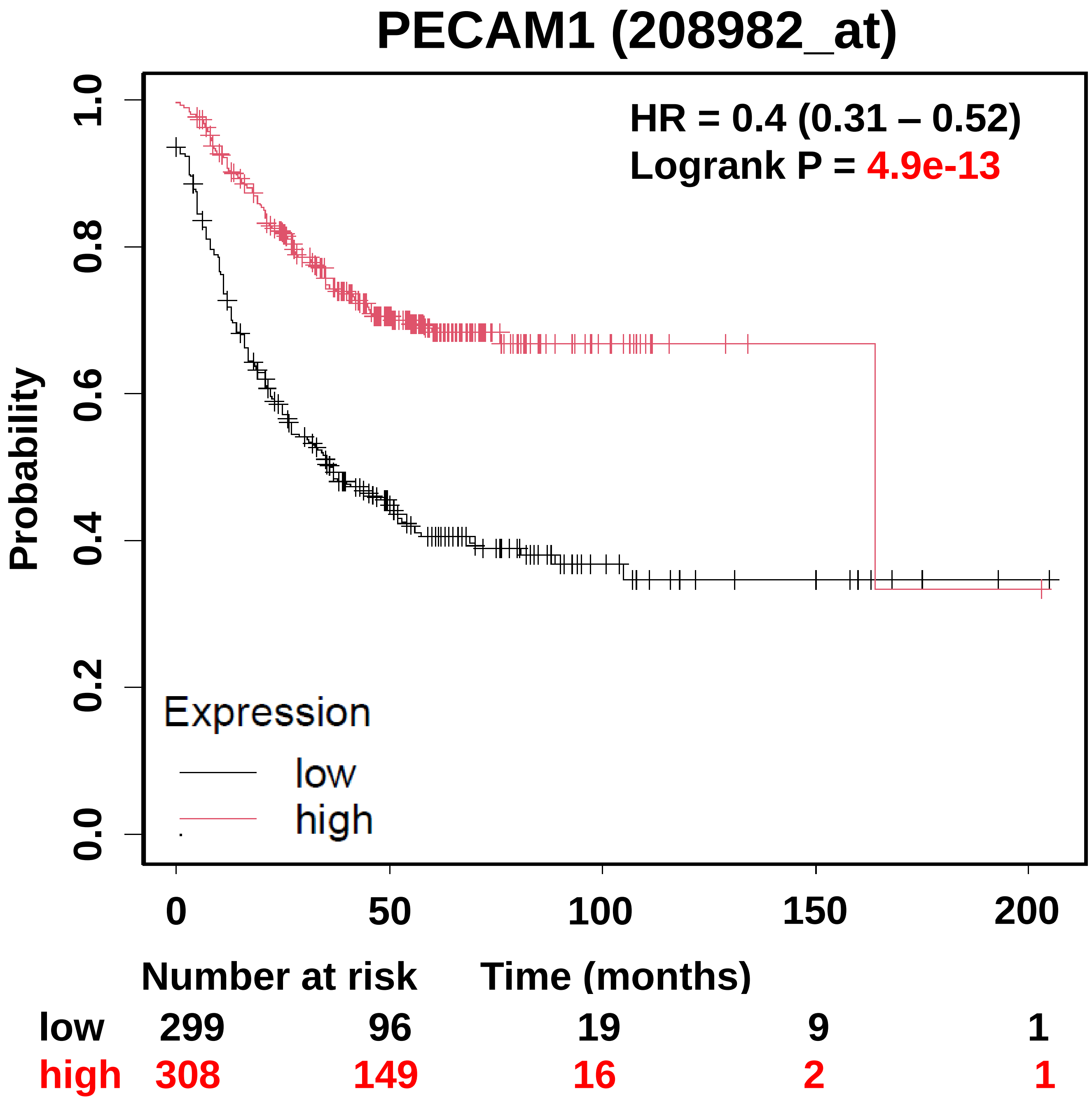
PECAM1 (208982_at)
HR = 0.4 (0.31 – 0.52)
Logrank P = 4.9e-13
Probability
0.0
0.2
0.4
0.6
0.8
1.0
Expression
low
high
0
50
100
150
200
Number at risk
Time (months)
low 299 96 19 9 1
high 308 149 16 2 1

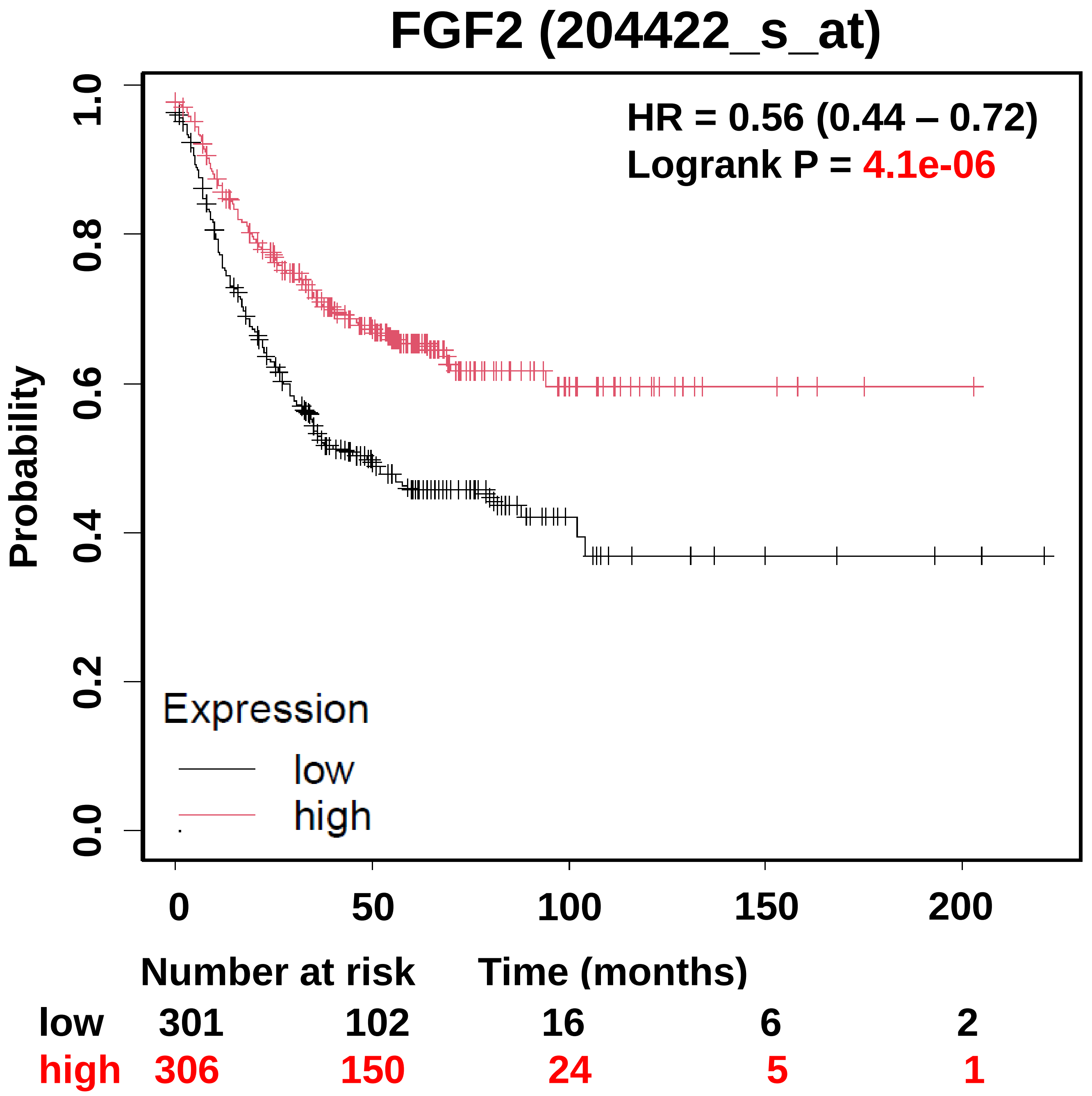
FGF2 (204422_s_at)
HR = 0.56 (0.44 – 0.72)
Logrank P = 4.1e-06
Probability
0.0
0.2
0.4
0.6
0.8
1.0
Expression
low
high
0
50
100
150
200
Number at risk
Time (months)
low 301 102 16 6 2
high 306 150 24 5 1

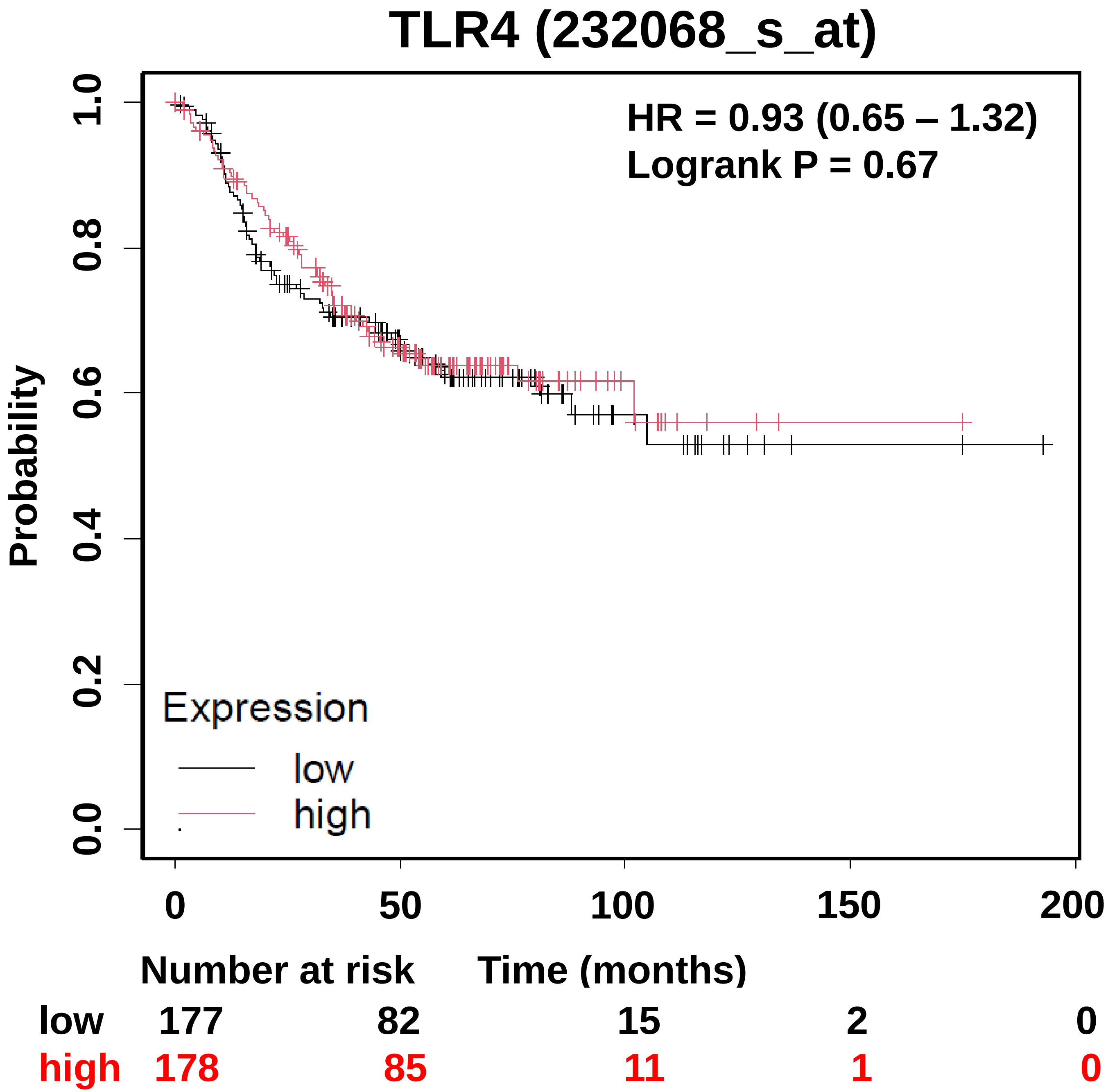
TLR4 (232068_s_at)
HR = 0.93 (0.65 – 1.32)
Logrank P = 0.67
Probability
1.0
0.8
0.6
0.4
0.2
0.0
Expression
low
high
0
50
100
150
200
Number at risk
Time (months)
low 177 82 15 2 0
high 178 85 11 1 0

**First progression survival (FP) analysis of hub genes in LUAD patients.** To avoid significance inflation associated with the KM-Plotter "auto select best cutoff," survival analyses were also proceeded to use predefined expression tertiles. For each hub gene, patients were split into T1 (low expression) and T3 (high expression) groups, and univariate Cox proportional hazards models were used to estimate HRs and 95% confidence intervals for overall survival (OS) and first progression (FP).
