# Supplementary material for: FGF2 as a Potential Tumor Suppressor in Lung Adenocarcinoma
Source: Diagnostics (Basel). 2026 Jan 13;16(2):250. doi: 10.3390/diagnostics16020250 (PMC12839716; doi:10.3390/diagnostics16020250)
Supplement: Supplementary file 1 [file diagnostics-16-00250-s001.zip › Supplementary Files S9.pdf]

Genetic Lab. Of TRI-I BIOTECH

TEST REPORT

ilac-MRA　TAF Testing Laboratory 2614

Report No.： CA2511210003
Contact Person： Ju-Fang Liu, PhD
Organization： School of Oral Hygiene, College of Oral Medicine, Taipei Medical University
Contact Address： No. 250, Wuxing Street, Taipei, Taiwan 111301, R.O.C.
Receiving Date： 2025/11/21
Test Date： 2025/11/24
Report Date： 2025/12/01
Test Item： Cell Line Authentication
Sample Type： □ Genomic DNA　■ Cell Pellet　□ FTA Card
Test Method： CA-TE01　Human cell DNA Extraction and Quantitation SOP
CA-TE02　GenePrint 24 System PCR and Genotyping SOP
CA-TE03　Human Cell STR data Quality Control and Analysis SOP

Tri-I Biotech, Inc. 源資生物科技檢驗實驗室 試驗報告專用章 新北市汐止區康寧街169巷25號5F

Approval Signatory：

Laboratory Head：

- This laboratory complies with ISO/IEC 17025:2017 and CNS 17025:2018. The data generated during the testing process fall within the quality control parameters, representing accurate and expected results.
- This test report is only responsible for the samples provided by the organization.
- This test report may not be excerpted or copied without the consent of this laboratory.

TRI Biotech 源資國際生物科技股份有限公司
221新北市汐止區康寧街169巷25號5樓
Tel: (02)-2695-4311 Fax: (02)-8695-1182
E-Mail: info@tri-ibiotech.com.tw
Website: www.tri-ibiotech.com.tw

Cell Line Authentication Report

Project NO: CA2511210003

Date:20251201

Customer: School of Oral Hygiene, College of Oral Medicine, Taipei Medical University

Sample name: CL1-0 and CL1-5

PCR amplification by GenePrint® 24 System

Genotyping analysis result:

Comparison with cell STR profile						
Marker	Test cell STR porfile			Test cell STR porfile		
	Sample name: CL1-0			Sample name: CL1-5		
	Allele1	Allele2	Allele3	Allele1	Allele2	Allele3
AMEL	X			X		
CSF1PO	10	13		13		
D10S1248	12	14	16	14		
D12S391	18	19	26	19		
D13S317	8	11		8		
D16S539	10	11	12	12		
D18S51	14			14		
D19S433	13	15.2		13	14	
D1S1656	14	15		14		
D21S11	32.2	33.2		32.2	33.2	
D22S1045	15			15		
D2S1338	17	19		17	19	
D2S441	10	11	12	10	12	
D3S1358	15			15		
D5S818	10	13		13		
D7S820	9	10	12	9		
D8S1179	14			13		
DYS391				10		
FGA	24			24		
Penta D	11			11		
Penta E	13	14	18	13		
TH01	7	9		9		
TPOX	8			8		
vWA	14	16	18	16	17	18

Biotech 源資國際生物科技股份有限公司

Are there multiple peaks that would be consistent with a mixture?	☐YES ■NO
Is the percent match result in the range 0-55 %?<br>This result is consistent with the two samples being unrelated (different donors)	■YES
Is the percent match result in the range 56-79 %?<br>This result is indeterminant and may need further testing	☐YES
Is the percent match result in the range 80-100 %?<br>This result is consistent with the two samples being related (same donor)	☐YES

*Percent Match = SHARED ALLELES x 2 / TOTAL ALLELES in the Test Sample + TOTAL ALLELES in the Reference Sample

The STR locus percent match of the CL1-0 and CL1-5 cell lines is below 80% in databases (**Cellosaurus**, **DSMZ**, and **ATCC**). There is no result in this test.

Similarity percentage: 69% (Between CL1-0 and CL1-5)

* Similarity percentage = SHARED ALLELES x 2 / TOTAL ALLELES in the Test Sample + TOTAL ALLELES in the Reference Sample

Tri-I Biotech, Inc.
TRI Biotech
源資生物科技核酸實驗室
試驗報告
專用章
新北市汐止區康寧街169巷25號5F

Operator: HSU CHUN YI

Lab manager: YA-WEN LEE

Tri-I Biotech, Inc.
Date:2025/12/01

Sample STR Genotyping Profile:

CL1-0:

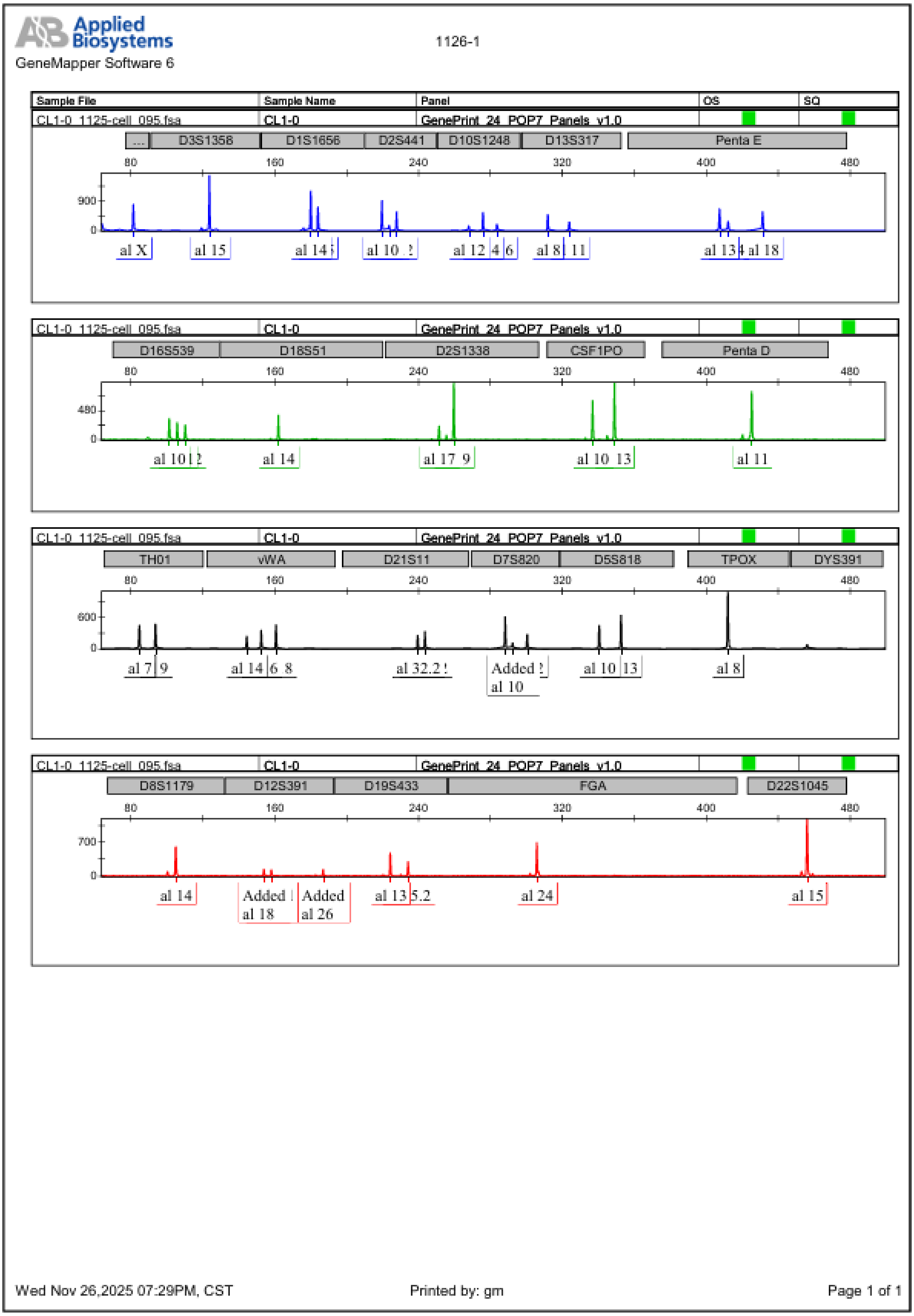

CL1-5:

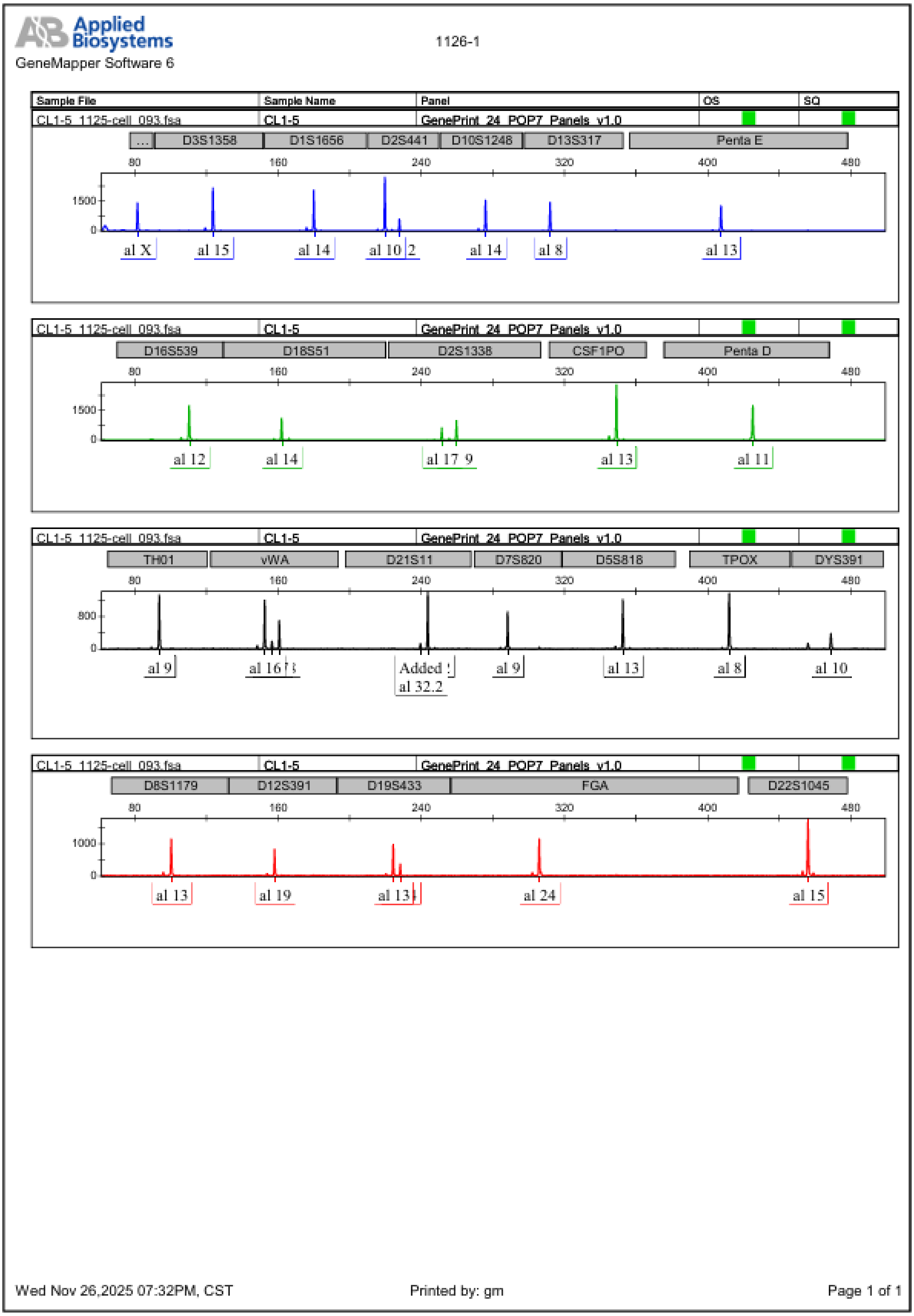

源資國際生物科技股份有限公司
TRI Biotech
