# Supplementary figures and images for: FGF2 as a Potential Tumor Suppressor in Lung Adenocarcinoma
Source: Diagnostics (Basel). 2026 Jan 13;16(2):250. doi: 10.3390/diagnostics16020250 (PMC12839716; doi:10.3390/diagnostics16020250)

### CL1-0

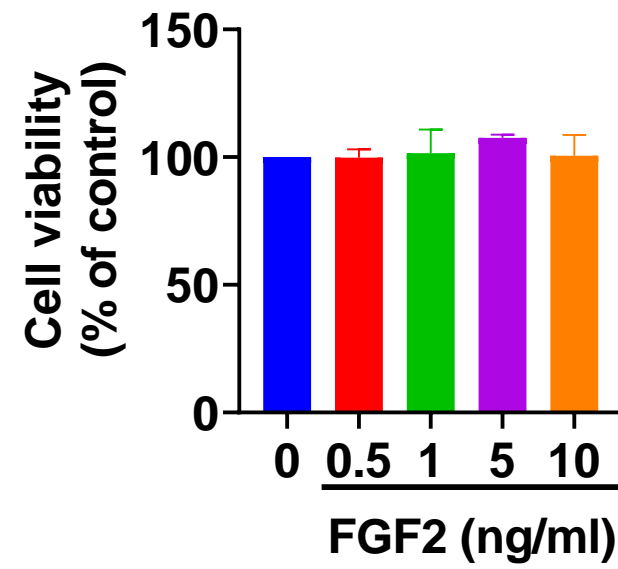

|                | O.D.   |        |        |        |
|----------------|--------|--------|--------|--------|
| Con            | 0.7294 | 0.7332 | 0.7184 | 0.7038 |
| FGF2 0.5 ng/ml | 0.7351 | 0.7273 | 0.6846 | 0.7314 |
| FGF2 1 ng/ml   | 0.7188 | 0.6691 | 0.827  | 0.7139 |
| FGF2 5 ng/ml   | 0.7634 | 0.7801 | 0.7845 | 0.7763 |
| FGF2 10 ng/ml  | 0.6571 | 0.7995 | 0.7323 | 0.7129 |

### CL1-5

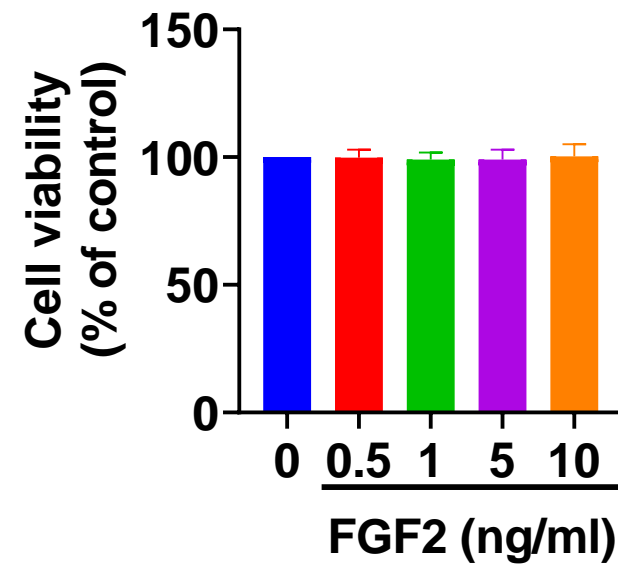

|                | O.D.   |        |        |        |
|----------------|--------|--------|--------|--------|
| Con            | 0.9581 | 0.9389 | 0.942  | 0.9423 |
| FGF2 0.5 ng/ml | 0.9264 | 0.9122 | 0.9785 | 0.9557 |
| FGF2 1 ng/ml   | 0.9293 | 0.9022 | 0.9528 | 0.9603 |
| FGF2 5 ng/ml   | 0.9615 | 0.8997 | 0.9736 | 0.9116 |
| FGF2 10 ng/ml  | 0.9651 | 0.9869 | 0.8833 | 0.9559 |

Supplement: Supplementary file 1 [file diagnostics-16-00250-s001.zip › Supplementary Files S8.pdf]
